# Roles and mechanisms of exosomal non-coding RNAs in human health and diseases

**DOI:** 10.1038/s41392-021-00779-x

**Published:** 2021-11-10

**Authors:** Chen Li, Yu-Qing Ni, Hui Xu, Qun-Yan Xiang, Yan Zhao, Jun-Kun Zhan, Jie-Yu He, Shuang Li, You-Shuo Liu

**Affiliations:** 1 Department of Geriatrics, The Second Xiangya Hospital, Central South University, Changsha, Hunan 410011 China; 2 Institute of Aging and Age-related Disease Research, Central South University, Changsha, Hunan 410011 China

**Keywords:** Predictive markers, Cancer, Endocrine system and metabolic diseases, Cardiovascular diseases, Neurodevelopmental disorders

## Abstract

Exosomes play a role as mediators of cell-to-cell communication, thus exhibiting pleiotropic activities to homeostasis regulation. Exosomal non-coding RNAs (ncRNAs), mainly microRNAs (miRNAs), long non-coding RNAs (lncRNAs), and circular RNAs (circRNAs), are closely related to a variety of biological and functional aspects of human health. When the exosomal ncRNAs undergo tissue-specific changes due to diverse internal or external disorders, they can cause tissue dysfunction, aging, and diseases. In this review, we comprehensively discuss the underlying regulatory mechanisms of exosomes in human diseases. In addition, we explore the current knowledge on the roles of exosomal miRNAs, lncRNAs, and circRNAs in human health and diseases, including cancers, metabolic diseases, neurodegenerative diseases, cardiovascular diseases, autoimmune diseases, and infectious diseases, to determine their potential implication in biomarker identification and therapeutic exploration.

## Introduction

Exosomes are a class of extracellular vesicles (EVs) ~30–150 nm in diameter.^1,2^ Exosomes were first reported in sheep reticulocytes in 1983. Further studies reported that exosomes are derived from most cell types and are present in the cell-conditioned medium and distinct biological fluids such as serum, plasma, urine, saliva, ascites, cerebrospinal fluid, and amniotic fluid.^3^ Exosomes were initially regarded as means of cellular waste disposing until further studies reported their role in mediating cell-to-cell communication, thus attracting significant attention of scholars worldwide.^4,5^ Several functions of exosomes have been characterized including cellular proliferation, differentiation, apoptosis, angiogenesis, and immune regulation.^6,7^ Exosomes exhibit these functions by interacting with surface receptors of recipient cells thus transmitting biomolecules such as lipids, proteins, messenger RNAs (mRNA), and non-coding RNAs (ncRNAs) to recipient cells.^8^ Notably, ncRNAs are the components of exosomes that have attracted particular attention.^9^

**Fig. 1:**
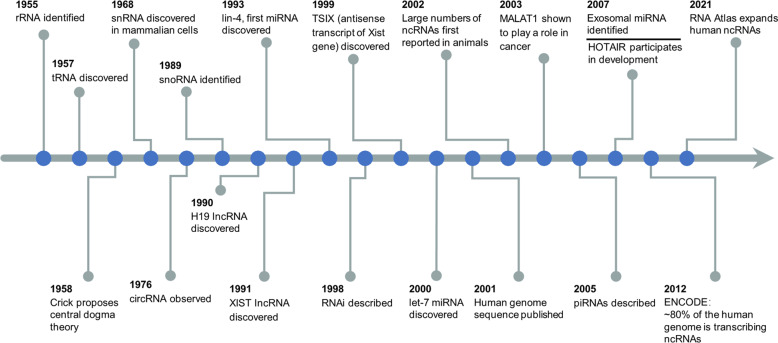
Timeline of the discovery and research history of ncRNAs in human health and diseases. Key discoveries are highlighted. XIST X (inactive)-specific transcript, RNAi RNA interference, piRNA PIWI-interacting RNA, HOTAIR HOX transcript antisense RNA, ENCODE Encyclopedia of DNA Elements

NcRNAs refer to molecules that lack protein-­coding regions, which have become a hot topic of increasing concern. The correlation of ncRNAs with human diseases has primarily been identified in the function and expression of miRNA found in cancers. However, the extent of ncRNAs involvement in diseases is only just being explored. To better understand the discovery and research history of ncRNAs in human health and diseases, it is helpful to review the timeline of ncRNAs (Fig. 1). Discovery of housekeeping ncRNAs, such as ribosomal RNA (rRNA)^10^ and transfer RNA (tRNA)^11^ in the 1950s, supported Crick’s “central dogma” theory, which stated that genetic information can proceed from DNA to RNA to protein.^12^ Further studies reported new ncRNAs, such as small nuclear RNAs (snRNA),^13,14^ small nucleolar RNAs (snoRNA)^15,16^ and circular RNAs (circRNAs).^17^ In the late 1980s, studies first reported long non-coding RNAs (lncRNAs), such as H19^18^ and Xist.^19,20^ The human genome sequence was published in 2001,^21^ and the findings showed that genes that encoded proteins only accounted for 1.2% of the genome, whereas the rest were considered as “non-coding”.^22^ The first small temporal RNAs, lin‑4,^23^ and let‑7,^24^ were discovered in Caenorhabditis elegans in 1993 and 2000, respectively. Analysis showed that ncRNA can act as conserved functional molecules needed for development. Further studies showed that large numbers of gene do not encode proteins but encode various unique transcripts.^25–28^ NcRNAs can regulate gene expression at transcriptional, post-transcriptional, and translational levels, thereby modulating associated signaling networks.^29^ In addition, different kinds of ncRNAs can interact with each other to regulate their stability or abundance.^30^ In 2007, it was reported that exosomes contain microRNAs (miRNAs) and mRNA and can transfer them to other cells.^31^ Since then, accumulating evidence demonstrates that a variety of ncRNAs can be encapsulated and transported by exosomes, the most attractive of which are miRNAs, lncRNAs, and circRNAs, explaining their roles in intercellular communication.^32,33^ Notably, exosomal ncRNAs exhibit diverse expression patterns in different cells or various physiological and pathological conditions, indicating the potential role of these exosomal biomolecules in occurrence and development of different diseases.^34,35^ These differences in expression levels in pathological states indicate that exosomal ncRNAs are promising diagnostic and therapeutic tools for various human diseases.^9,36–38^ A large number of studies dealing with circulating exosomes and their cargoes prove that exosomal miRNAs, lncRNAs and circRNAs are closely involved in human health and the initiation and development of various diseases.^39^ Therefore, we specifically focus on effects of exosomal ncRNAs (miRNAs, lncRNAs, and circRNAs) in physiopathology, clinical diagnosis, and therapy of human diseases, such as metabolic diseases, cancers, neurodegenerative diseases, cardiovascular diseases, autoimmune diseases, and infectious diseases.

## Potential regulatory mechanisms of exosomes in human diseases

**Fig. 2:**
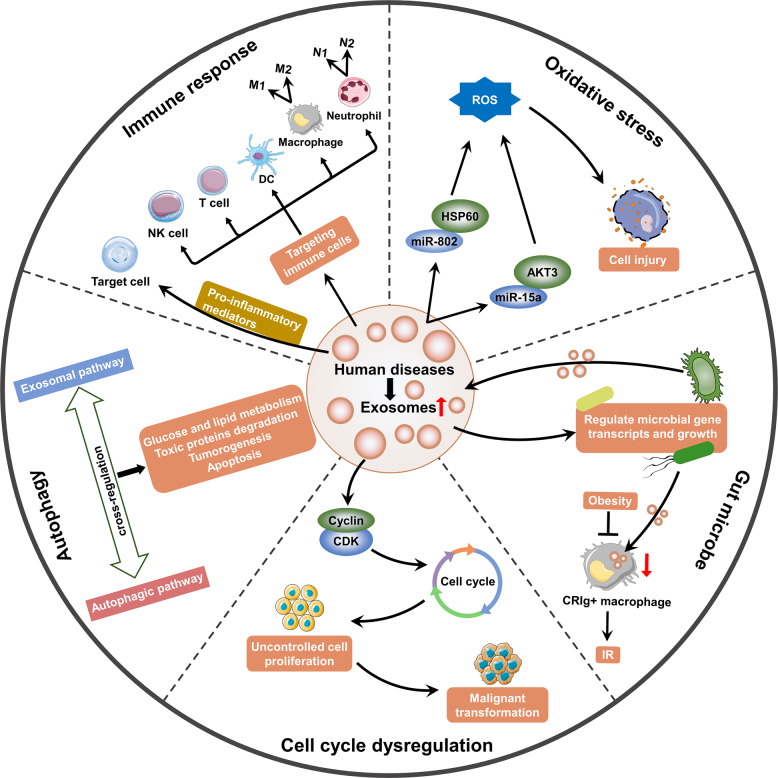
The potential regulatory mechanisms of exosomes in human diseases. Several mechanisms of the occurrence of human diseases are modulated by exosomes, including immune response, oxidative stress, autophagy, gut microbe, and cell cycle. This figure was created with the aid of Servier Medical Art (https://smart.servier.com/). ROS reactive oxygen species, HSP heat shock protein, AKT3 AKT serine/threonine kinase 3, IR insulin resistance, CDK cyclin-dependent kinase, NK natural killer, DC dendritic cell

Several studies reported the importance of exosomes in human health and diseases.^40,41^ In addition, recent studies demonstrated that exosomes could exert their roles by modulating immune response, oxidative stress, autophagy, gut microbe, and cell cycle.^42–45^ This section provides a comprehensive overview of the current understanding of exosomes research and discusses potential mechanisms of exosomes in human diseases (Fig. 2).

### Immune response

Exosomes released from immune and non-immune cells exert a pivotal effect in immune regulation.^46–48^ Recent studies reported the functions of exosomes in triggering or inhibiting immune response, indicating their potential roles in the development and progression of autoimmune and inflammatory diseases.^49–54^

Exosomes can regulate immune response via the transfer and presentation of antigenic peptides. Exosomes derived from antigen-presenting cells (APCs) activate T cells by carrying major histocompatibility complex class II (MHC-II) that binds to antigenic peptides.^48^ Notably, APC-released exosomes with MHC-II bearing tumor peptides, significantly inhibit tumor growth in mice in a T cell-dependent manner.^55^ In addition, exosomes from APCs carrying bacterial antigens promote activation of anti-bacterial immunity. For instance, macrophage-derived exosomes carrying mycobacterial antigens protect mice against mycobacterium tuberculosis infection by inducing CD4^+^ and CD8^+^ T cells to produce IFN-γ and IL-2.^56^ However, excessive immune response mediated by exosomes can cause normal tissue damage thus promoting onset and development of diseases. For example, circulating exosomes from patients with Hashimoto thyroiditis can present antigens to dendritic cells (DCs), thus inducing DC activation through the NF-κB signaling pathway, contributing to imbalanced differentiation in CD4^+^ T cells, and potentially leading to Hashimoto thyroiditis onset.^54^

Exosomes can mediate tumor or pathogen immune escape by affecting gene expression in immune cells, mainly by delivering miRNAs, thus promoting progression of cancers and infection. For instance, tumor-derived exosomal miR-212-3p downregulates expression of regulatory factor X-associated protein (RFXAP), which inhibits MHC-II and promotes immune tolerance of dendritic cells.^57^ Moreover, virus-infected cell can transmit viral miRNAs to uninfected host immune cells through exosomes, thus downregulating immunomodulatory genes.^58^ Therefore, exosomes present a novel intersection between immune response and disease.

Moreover, exosomes seem to be involved in the regulation of macrophage and neutrophil polarization, which ultimately can induce the pathophysiologic process of several diseases. Macrophages comprise a population of heterogeneous cells which are classified into two classes including pro-inflammatory M1 or anti-inflammatory M2 macrophages based on their activation status.^59^ Exosomes can induce macrophage differentiation into M1 or M2 phenotypes, a critical regulatory mechanism of inflammation, which has essential effects on homeostasis.^60–63^ For example, adipose-derived exosomes exacerbate insulin resistance (IR) and atherosclerosis by inducing macrophage M1 polarization.^62,64^ Moreover, exosomes derived from cancer cells can also promote M1 macrophage polarization. Xiao and coworkers reported that exosomal thrombospondin-1 (THBS1) secreted by oral squamous cell carcinoma cells can activate M1 macrophages polarization to promote malignant migration.^65^ Notably, exosomes can induce activation of M2 macrophages thus inhibiting inflammatory response, leading to abrogation of many diseases.^66,67^ Studies reported that exosomes from mesenchymal stem cells (MSCs) ameliorate cardiac damage in myocardial infarction rats and ischemia/reperfusion mice by activating macrophage M2 polarization.^68,69^ However, M2 macrophages polarization can be detrimental. For example, M2 macrophages support tumor growth and survival.^70^ Studies report that tumor-derived exosomes promote occurrence and development of tumor by activating M2 phenotypes. Exosomal DLX6-AS1 from HCC cells triggers M2 macrophage polarization to provoke tumor invasion and migration through the miR-15a-5p/CXCL17 axis.^71^ Besides, exosomes can play a crucial role in disease progression by inducing neutrophil polarization.^72,73^ Laboratory tests have confirmed the existence of N1 (antitumoral) and N2 (protumoral) tumor-related neutrophils, parallel to M1 and M2 macrophage polarization.^74^ Moreover, tumor-derived exosomes can induce N2 polarization of neutrophils to promote cancer progression.^72^ Conversely, exosomes that inhibit neutrophil inflammatory response can alleviate the tissue injury and have the therapeutic potential.^75^

In addition, exosomes can modulate immune response by transporting cytokines or other pro-inflammatory mediators thus directly acting on target organs.^76–78^ Macrophage-derived exosomes containing miR-21-5p promote inflammatory activation and regulate podocyte injury in diabetic nephropathy mice.^79^ Fabbri et al. found that tumor-secreted exosomal miRNAs induce inflammatory response that may contribute to tumor growth and metastasis.^80^ Conversely, some exosomes exert anti-inflammatory effects that can be targeted for development of therapies.^81^ For instance, exosomal miR-192 significantly attenuates tumor metastasis by suppressing secretion of proangiogenic factors, such as interleukin (IL)-8, intercellular cell adhesion molecule (ICAM), and C-X-C motif chemokine ligand 1(CXCL1).^82^

Collectively, exosomes have been identified to regulate immune responses by carrying biomolecules to targeted cells, thereby affecting the phenotype and immunomodulatory functions of immune cells, or directly acting on target organs. Particularly, exosomes derived from immune cells or non-immune cells exert pivotal roles in immunotherapy. In this section, we discuss the roles of exosomes as carriers for regulating immune responses and as predictive biomarkers for immune activation.

### Oxidative stress

Oxidative stress refers to an imbalance in oxidative-antioxidative systems and leads to excessive accumulation of reactive oxygen species (ROS), which contributes to various disorders by inducing cell and tissue dysfunction.

Nutrition stress promotes oxidative stress.^83^ Advanced glycation end-products (AGEs) play an important role in ROS production and promote oxidative stress in diabetes individuals.^84^ Exosomes exhibit an essential role in oxidative stress. Exosomal miR-802-5p derived from hypertrophic adipocyte causes cardiac IR by suppressing expression of heat shock protein 60 (HSP60), which is implicated in promoting oxidative stress.^85^ Kamalden and coworkers reported that pancreatic β-cells-derived exosomal miR-15a can travel through the circulation and induce oxidative stress by targeting AKT serine/threonine kinase 3 (AKT3), leading to retinal injury in T2DM subjects.^43^ Furthermore, exosomal-miR-21-5p from macrophages induces podocyte injury in diabetic nephropathy mice partially by promoting ROS production.^79^ In addition, oxidative stress is associated with age-related bone loss that increases the risk of osteoporosis.^86^ Exosomes from serum of aged normal individuals can exert a protective effect on bone health by inhibiting aging-related oxidative stress.^87^ Recent studies have documented that exosomal circHIPK3 produced by hypoxia-pretreated cardiomyocytes decreases oxidative stress-induced cardiac microvascular endothelial cells dysfunction.^88^

To sum up, these findings indicate the relationship between exosomes and oxidative stress in human disease. Exosomes have been shown to play a vital part in modulation of oxidative stress during the occurrence and development of various diseases.

### Autophagy

Under normal physiological conditions, autophagy serves as a protective mechanism to remove protein aggregates, impaired organelles, and invading pathogens, and is implicated in recycling amino acids, lipids, and sugars to maintain cellular renovation and homeostasis.^89,90^ However, autophagy dysfunction is associated with several diseases, such as cancers, neurodegenerative diseases, and metabolic diseases. The crosstalk between autophagy and exosome biogenesis varies with the type of disease.^91^ Autophagy can reduce release of exosomes through multivesicular bodies (MVB) degradation.^92^ In addition, exosome release and autophagy can act synergistically against cell stress.^93^

Reduced autophagy level is reported in multiple diseases, such as obesity, hyperglycemia, and osteoporosis.^90,94–96^ Li and colleagues revealed that defective autophagy induced by adipose sirtuin1 (SIRT1) deficiency increases exosome release from adipose tissue, thus promoting decreased glucose tolerance, diminished insulin sensitivity, and impaired lipid metabolism.^97^ Mammalian sterile 20-like kinase 1 (Mst1)-enclosed exosomes from cardiac microvascular endothelial cells (ECs) are implicated in inhibition of autophagy, promotion of apoptosis and suppression of glucose uptake in diabetic cardiomyopathy.^98^ Notably, exosomes play a cell protective role by activating intracellular autophagy.^91^ MSC transplantation after myocardial infarction improves cardiac function and infarct size partially through release of exosomes that improve autophagic flux. Accumulating evidence indicates that MSC-derived exosomes alleviate T2DM complications including diabetic ulcers and nephropathy by inducing autophagy.^44,99–101^ A similar effect is reported in exosomes released from M2 macrophage.^102^

Neuronal cells can eliminate protein aggregates to ameliorate proteotoxicity through autophagic degradation and exosome release. Conversely, abnormal accumulation and aggregation of proteins are manifestations of various neurodegenerative diseases. Exosome secretion can be elevated to attenuate the toxic proteins during autophagic or lysosomal dysfunction. For instance, Yang and coworkers identified secretory carrier membrane protein 5 (SCAMP5) as an autophagy inhibitor that promote exosomal secretion of alpha-synuclein (α-SYN).^103^

Studies report that cancer cell-derived exosomes affect autophagy in recipient cells.^104^ In addition, exosome regulate drug resistance and tumor microenvironment in an autophagy-dependent manner.^105^ For instance, exosomal circ-PVT1 promotes cisplatin resistance in gastric cancer cells by inducing cell autophagy and invasion and inhibiting apoptosis.^106^ Furthermore, gastric cancer cell-derived exosomes trigger autophagy and promote activation of neutrophils, ultimately promoting gastric cancer cell migration.^72^

Altogether, these findings indicate that exosomes play important roles in multiple physiological and pathological processes by regulating autophagy. Besides, the biogenesis and release of exosomes are closely associated with autophagy in diseases. Autophagy dysfunction is one of the important potential mechanisms of exosomes in many diseases, such as metabolic diseases, neurodegenerative diseases, and cancers.

### Gut microbe

Gut microbes exert important roles in physiological processes, such as providing essential nutrients, assisting in cellulose digestion, regulating integrity of gut barriers and immune response.^107^ However, dysbiosis, the imbalance in microbiota composition and diversity, in response to internal changes or external stimuli is associated with several chronic diseases, such as autoimmune, metabolic, cardiovascular diseases, and cancer.^108–110^ Intestinal homeostasis relies on dynamic and coordinated interactions between microbes, epithelium, and host immune system. Accumulating evidence has supported that exosomes provide a link between the host and gut microbial community. Liu and coworkers uncovered a new effect of fecal exosomal miRNAs on shaping gut microbiota. Intestinal ECs-derived miRNAs-containing exosomes can enter bacteria and regulate gene transcripts and growth, and their loss leads to dysbiosis and aggravation of colitis.^111^ These findings indicate the key roles of fecal exosomal miRNAs on maintaining normal gut microbiota.

Exosomes from beneficial microbes can improve metabolic functions through various mechanisms. Recent studies report that exosomes can restore intestinal and metabolic homeostasis in HFD-induced obesity mice by reversing the adverse effects of obesity including adipose and gut inflammation, intestinal mucosal barrier permeability, and fat weight gain.^45,112^ Conversely, maleficent bacteria is implicated in impairing of metabolic homeostasis. Stool exosomes from *Pseudomonas panacis* in HFD mice promote glucose intolerance and IR in healthy mice.^113^ Liver CRIg^+^ (complement receptor of the immunoglobulin superfamily) macrophage can clear bacteria and their products from the bloodstream. A recent study reported a decrease in CRIg^+^ macrophage population in obese subjects. As a result, less circulating gut microbial DNA-containing exosomes were eliminated, and more exosomes diffused to distant metabolic tissues, thus aggravating tissue inflammation and IR.^114^ In addition, gut microbiota are involves in modulation of bone metabolism. A recent study reported that gut microbiota in children and *Akkermansia muciniphila* release exosomes to bone tissues to ameliorate osteoporosis via promoting osteogenic activity and decreasing osteoclast formation.^115^

With the relevant cumulative findings, we summarize and indicate the role of exosomes as a link between gut microbiota and diseases. Moreover, these findings suggest that the effects of exosomes on the microbiome may be utilized to target specific host processes to ameliorate diseases.

### Cell cycle dysregulation

Cell proliferation and division are basic cell physiological activities. Growth factors, hormones, and oncogene products can induce or inhibit cell proliferation, thereby influencing the cell cycle. Dysregulation of the cell cycle is associated with multiple diseases. Accelerated cell cycle can lead to carcinogenesis.^116^ Recent studies have revealed the essential role of exosomes in regulating cell cycle. Exosomal circRNA_100284 accelerates cell cycle and promotes proliferation by targeting miR-217, which induces malignant transformation of human hepatic cells.^117^ In addition, exosomal lncRNA ZFAS1 promotes gastric cancer progression by shortening cell cycle and epithelial-mesenchymal transition (EMT).^118^ Conversely, exosomes that block cell cycle in cancer can serve as therapeutic targets. ADSC-released exosomal-miRNAs present inhibitory effects on ovarian cancer cells by blocking cell cycle and inducing apoptosis signaling.^119^ These findings show the dual effects of exosomes on cell cycle regulation in the initiation and progression of cancers.

### Differences of exosomal and non-exosomal ncRNAs in health and diseases

**Fig. 3:**
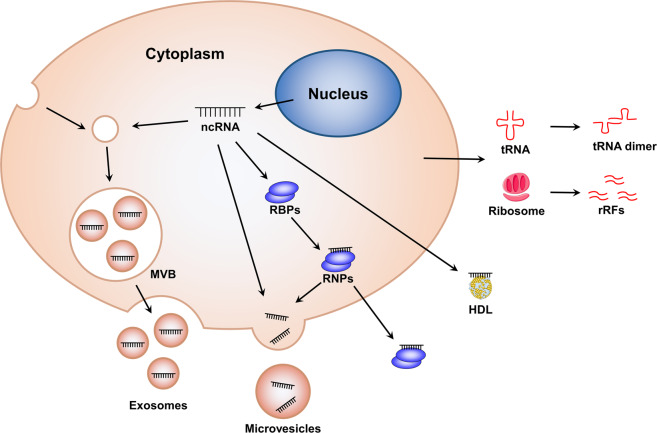
Different mechanisms underlying the stability of extracellular ncRNAs. NcRNAs can be protected from harsh extracellular environment through extracellular vesicles encapsulation (such as exosomes and microvesicles), ribonucleoprotein (RNP) complex formation, and high-density lipoprotein (HDL) transportation. Moreover, some extracellular RNA fragments that generate from non-vesicular ncRNAs in extracellular space can form self-protecting dimers. The source of these non-vesicular RNAs remains uncertain. This figure was created with the aid of Servier Medical Art (https://smart.servier.com/). MVB multivesicular bodies, RBPs RNA-binding protein, rRFs rRNA-derived fragments

It has been confirmed that ncRNAs exist not only in cells, but also in different body fluids such as serum, plasma, urine, saliva, and so on. The ncRNAs in biofluids are often referred to as extracellular ncRNAs or circulating ncRNAs. Notably, the RNase activity is high in the extracellular environment, but extracellular ncRNAs remain relatively stable in plasma, suggesting that circulating ncRNAs may be protected and circumvented from harsh conditions. One intriguing mode of transport of circulating ncRNAs is related to exosomes. In this section, we try our best to investigate the differences between ncRNAs in exosomes and non-exosomes in regulating physiological homeostasis and pathological processes in health and diseases (Fig. 3).

NcRNAs can be encapsulated by EVs (including exosomes, microvesicles, and apoptotic bodies) and secreted out of cells to act as mediators for intercellular communication, thereby regulating different diseases according to the target cells. In addition to the vesicle-dependent pattern, a considerable number of extracellular ncRNAs are present in the form of ribonucleoprotein (RNP) complexes with RNA-binding proteins (RBPs) such as argonaute-2 (AGO2) that modulate mRNA inhibition in cells.^120,121^ These RBPs can affect RNA sorting into EVs in an indirect manner.^122^ Moreover, high-density lipoprotein (HDL) can also transport endogenous ncRNAs to recipient cells with functional targeting capabilities, the effect of which may vary depending on the disease state.^123^ Further, recent studies have revealed a post-release processing of ncRNAs. Some extracellular RNA fragments that generate from non-vesicular ncRNAs, such as ribosomes and full-length tRNAs, in extracellular space can form self-protecting dimers to resist RNases.^124,125^

## The roles of exosomal ncRNAs in human diseases

**Fig. 4:**
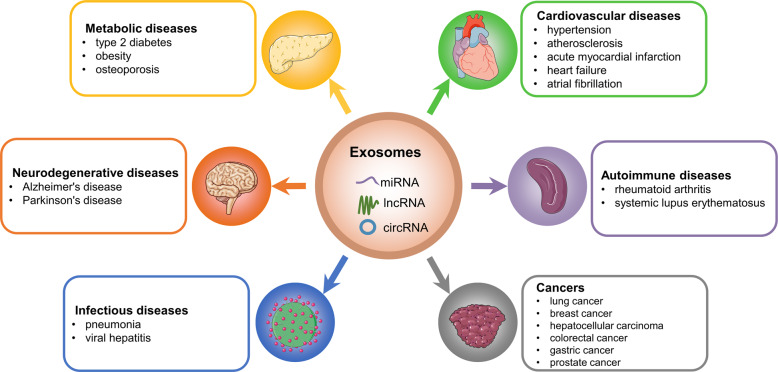
The roles of exosomal ncRNAs in human diseases. The figure showed examples of human diseases where exosomal ncRNAs exert pivotal function. This figure was created with the aid of Servier Medical Art (https://smart.servier.com/)

Accumulating data suggest that exosomal ncRNAs exert pleiotropic effects on human diseases.^126,127^ Among these ncRNAs cargoes, the most intriguing ones are miRNAs, lncRNAs, and circRNAs. MiRNAs are small, highly conserved ncRNAs.^128^ LncRNAs are poorly conserved ncRNAs with a length of more than 200 nucleotides.^30^ CircRNAs are a subset of ncRNAs with covalently closed structures which are implicated in the regulation of gene expression.^129^ In this section, the roles of exosomal miRNAs, lncRNAs, and circRNAs in different human diseases (including cancers, metabolic diseases, cardiovascular diseases, neurodegenerative diseases, autoimmune diseases, and infectious diseases) are explored (Fig. 4).

### The roles of exosomal ncRNAs in cancers

**Fig. 5:**
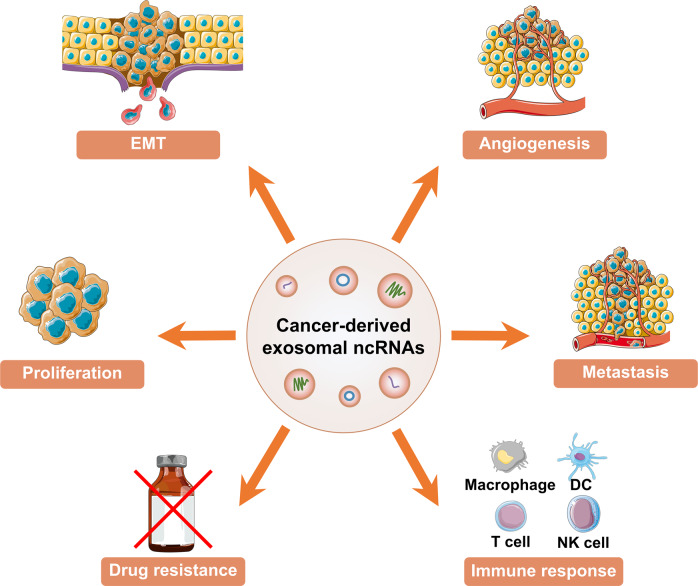
The roles of exosomal ncRNAs in cancer. Exosomal ncRNAs play a role in cancers, including EMT, proliferation, angiogenesis, metastasis, drug resistance, and immune response. This figure was created with the aid of Servier Medical Art (https://smart.servier.com/). EMT epithelial-mesenchymal transition, DC dendritic cell, NK natural killer

GLOBOCAN statistics report that approximately 14.1 million new cancer cases were diagnosed and 8.2 million deaths occurred in 2012. Prevalence of cancer is raising owing to the increase in population growth and aging population, creating a huge health burden for both patients and society.^130^ Recent studies have explored tumor-associated exosomal ncRNAs.^131^ Emerging studies revealed that exosomal ncRNAs are implicated in progression of human cancers, such as lung cancer,^132^ breast cancer (BC),^133^ and hepatocellular carcinoma (HCC).^134^ Exosomal ncRNAs play a role in cancers, including EMT, proliferation, angiogenesis, metastasis, drug resistance, and immune-inflammation (Fig. 5). In this section, the roles of exosomal miRNAs, lncRNAs, and circRNAs in various human cancers, including lung cancer, BC, HCC, colorectal cancer (CRC), gastric cancer (GC), and prostate cancer (PCa) are summarized.

#### Exosomal ncRNAs in lung cancer

Lung cancer is the leading cause of cancer-related mortality globally. Approximately 70% of lung cancer patients present with complex complications at the time of diagnosis and surgical resection is the primary treatment option for lung cancer.^135^ Association of exosomal ncRNAs and lung cancer can be explored to identify novel biomarkers for tumor targeted diagnosis and therapy.^132^ Numerous lines of publications have indicated that exosomes and exosomal ncRNAs exert important roles in multiple cellular and molecular processes linked to lung cancer, thus providing new diagnostic biomarkers and therapeutic targets in lung cancer.

EMT is tightly associated with tumor invasion and metastasis by promoting lung cancer cells infiltration and migration.^136^ Kim et al. reported that miR-23a is significantly enriched in TGF-β1-treated human lung adenocarcinoma (LUAD) cells and is involved in EMT.^137^ Notably, bone marrow-derived mesenchymal stem cells (BMSCs) are essential components of cancer microenvironment and are involved in development of lung cancer. Zhang et al. demonstrated that BMSCs-derived exosomal miR-193a-3p, miR-210-3p, and miR-5100 promote lung cancer cells invasion by activating signal transducer and activator of transcription 3 (STAT3) signaling pathway and triggering EMT.^138^ Furthermore, miR-499a-5p is upregulated in highly metastatic cells-derived exosomes and enhances cell proliferation, migration, and EMT by targeting the rapamycin (mTOR) pathway.^139^

Cellular proliferation and migration are implicated in cancer onset and development.^140^ It has been reported that exosomal miR-96 plays a role in lung cancer cells proliferation and migration by targeting LIM-domain only protein 7 (LMO7).^141^ Non-small cell lung cancer (NSCLC) accounts for approximately 85% of all lung cancer cases.^142^ Exosomes released from gemcitabine-resistant A549 cells can transfer miR-222-3P to target cells and promote cell proliferation, migration, and invasion by targeting suppressor of cytokine signaling 3 (SOCS3).^143^ On the contrary, EC-derived exosomes are characterized by high level of miR-126 and play a role in inhibiting cell proliferation and reducing loss of malignancy of NSCLC cells.^144^ Mechanistically, exosomal miR-126 suppress NSCLC development by targeting insulin receptor substrate 1 (IRS1) and vascular endothelial growth factor (VEGF). A549 NSCLC cells-derived exosomal miR-208a inhibits NSCLC cell proliferation by targeting p21 thus activating the AKT/mTOR pathway.^145^ In addition, exosomal miR-512 suppresses cell proliferation by targeting TEA domain family member 4 (TEAD4).^146^

Oxygen and nutrients are necessary for survival of cancer cells, thus angiogenesis is essential for tumor growth and metastasis.^147^ Zhuang et al. reported that exosomal miR-9 enhances angiogenesis by activating JAK/STAT pathway.^148^ Moreover, human bronchial epithelial (HBE) cells-derived exosomal miR-21 can promote angiogenesis by activating STAT3 and increasing expression of VEGF.^149^ Furthermore, circulating exosomal miR-23a levels are positively correlated with lung cancer proangiogenic activities. Hypoxic lung cancer cells promote angiogenesis by repressing the tight junction protein ZO-1 through exosomal miR-23a.^150^ Exosomal miR-126 secreted by NSCLC cells can trigger angiogenesis and accelerate lung cancer progression.^144^ In addition, miR-210 packaged in exosomes from tumor cells stimulates angiogenesis.^151^

Metastasis is a primary feature of cancer. Bone metastasis is common in patients with lung cancer.^131^ MiR-21 in A549 cells-derived exosomes promotes tumorigenesis and osteoclastogenesis by targeting the PDCD4 pathway.^152^ In addition, immune responses are involved in cancer progression. Fabbri et al. demonstrated that NSCLC cells-derived exosomes are characterized by high expression levels of miR-21, miR-27b, and miR-29a. Notably, exosomal miR-21 and miR-29a can promote tumor growth and metastasis by targeting Toll-like receptor (TLR) and inducing pro-metastatic inflammatory responses.^80^ Moreover, overexpression of exosomal miR-192 significantly appeased osseous metastasis by suppressing secretion of proangiogenic factors.^82^

Exosomal lncRNAs have been reported to be highly correlated with lung cancer. Exosomal lncRNAs such as H19, MALAT1, HOTAIR, UCA1, lnc-MMP2-2, GAPLINC, TBILA, AGAP2-AS1, and SOX2-OT play several roles in pathological processes including cell proliferation, migration, invasion, and EMT linked to lung cancer.^153^ Li et al. reported that GAS5 in exosomes is not only important for cancer development, but a promising biomarker for diagnosis patients with early NSCLC.^154^ Teng et al. found that exosomal SOX2-OT can be used as an effective noninvasive plasma-based tumor marker for lung squamous cell carcinoma (LSCC).^155^

Exosomal circRNAs are involved in the development of lung cancer. CircSATB2 are enriched in NSCLC cells and can be transported to other cells by exosomes to facilitate cell proliferation, migration, and invasion of NSCLC cells, and trigger abnormal proliferation of bronchial epithelial cells.^156^ Exosomal has-circ-0014235 promotes NSCLC progression by targeting miR-520a-5p/cyclin-dependent kinase 4 (CDK4) axis.^157^ Besides, circRNA-002178 shuttled by exosomes can be transferred to CD8^+^ T cells thus promoting generation of programmed death-ligand 1 (PDL1)/programmed cell death protein 1 (PD1) in LUAD.^158^ In addition, the expression levels of exosomal circ-0007761, circ-0047921, circ-0056285, circ-0008928, circRNA-102481, circ-MEMO1, circ-ARHGAP10, circ-PIP5K1A, and circ-FARSA are changed in NSCLC,^159–165^ while that of exosomal circ-0000690, circ-0001346, circ-0001439, and circ-0001492 are significantly increased in LUAD, which have potential as promising diagnostic biomarkers.^166^

#### Exosomal ncRNAs in BC

BC is the most frequent female malignant tumor globally and 70–80% patients present with early, non-metastatic BC which is considered curable.^167^ Approximately 2.1 million cases of BC were diagnosed in women and 626,679 breast cancer-related deaths were reported in 2018.^168^ Over the past decades, increasing evidence has shown that exosomal ncRNAs are closely associated with BC development.^169^

Exosomal miRNAs play roles in cellular proliferation, migration, and invasion of BC. A previous study reported that exosomal miR-10b is highly expressed in BC cells and promotes invasion by inhibiting expression of HOXD10 and KLF4.^170^ Exosomal miR-1246 is highly expressed in breast cancer cells and enhances cell proliferation and invasion by targeting CCNG2.^171^ On the contrary, exosomal miR-134 downregulates and inhibits cell proliferation, migration and invasion by targeting STAT5B in BC.^172^ Overexpression of miR-130a-3p in BC stem cells inhibits cellular proliferation, migration, and invasion by targeting RAB5B.^173^ In addition, miR-127, miR-197, miR-222, and miR-223 shuttled by exosomes inhibits cell proliferation by suppressing CXCL12.

Exosomal miRNAs are also involved in metastasis of BC. Cancer-associated fibroblasts (CAFs) are essential components of tumor microenvironment and play important roles in tumor development and metastasis. Exosomal miR-9 is highly expressed in breast CAFs and promotes switch of fibroblasts to CAF phenotype.^174^ Wu et al. revealed that exosomal miR-16 and miR-148a from focal adhesion kinase knockout CAFs are upregulated and ameliorate tumor cell metastasis.^175^ CAFs-derived exosomal miR-21, miR-143, and miR-378e promote stemness and EMT phenotype of BC cells.^176^ Moreover, miR-1910-3p shuttled by exosomes promotes cell proliferation and metastasis by targeting MTMR3 and NF-κB signaling pathway.^104^ In addition, exosomal miR-503-3p, miR-4269, miR-30e-3p, miR-105, miR-122, miR-200, miR-939, and miR-940 play crucial roles in promoting BC progression and metastasis.^177–182^

Apart from these, exosomal miRNAs participate in other cellular process of BC. For instance, BC cells-derived exosomal miR-20a-5p enhances differentiation of osteoclasts by targeting SRCIN1.^183^ MiR-23b is upregulated in BC cells-derived exosomes and reduces expression of MARCKS, a key regulator of cell cycling and motility.^184^ Notably, miR-210 from BC cells-derived exosomes can promote angiogenesis.^185^ On the contrary, miR-16 and miR-100 in exosomes from mesenchymal stem cells (MSCs) reduce secretion of VEGF in tumor cells and inhibit angiogenesis.^186,187^

Exosomal lncRNAs play key roles in BC progression and are potential diagnostic biomarkers for BC. LncRNA MALAT1 shuttled by BC exosomes can promote cell proliferation.^188^ Exosomal lncRNA GS1-600G8.5 are highly expressed in brain metastatic breast cancer cells and are implicated in destroying the BBB system and promoting transfer of cancer cells across the BBB.^189^ Moreover, expression of exosomal lncRNAs, including H19, SUMO1P3, XIST, and HOTAIR is upregulated in patients with BC indicating that they can serve as promising diagnostic biomarkers for BC.^190–194^

Exosomal circRNAs exhibit various roles in BC. Yang et al. found that serum exosomal circPSMA1 from BC is highly upregulated and promotes BC tumorigenesis, migration, and migration through miR-637/Akt1/β-catenin (cyclin D1) axis.^195^ Besides, circHIF1A (circ-0032138) is highly expressed in hypoxic CAFs-derived exosomes and modulates stem cell properties of BC through miR-580-5p/CD44 axis.^196^ Besides, exosomal circHIF1A significantly promotes BC growth and metastasis by activating AKT/STAT3 signaling pathway and suppressing expression of P21.^197^ On the contrary, Wang et al. reported that expression of several exosomal circRNAs was downregulated in BC cells.^198^

#### Exosomal ncRNAs in HCC

HCC is a major cause of cancer-related deaths worldwide.^199^ Notably, HCC is commonly diagnosed in cirrhosis patients.^200^ Recent studies have explored the biological functions of exosomal ncRNAs in initiation and development of HCC and report that exosomal ncRNAs can be used as non-invasive biomarkers for HCC.^201^

Exosomal miRNAs are closely associated with the pathology of HCC. Cui et al. reported that exosomal miR-224 promotes cell proliferation by targeting glycine N-methyltransferase.^202^ In addition, exosomal miR-93 can stimulate proliferation and invasion of HCC by suppressing TIMP2/TP53INP1/CDKN1A.^203^ Moreover, HCC cell-derived exosomal miR-665 promotes tumor cell proliferation by targeting MAPK/ERK signal pathway.^204^ Studies report that serum exosomal miR-1247-3p is implicated in lung metastasis in HCC patients. Exosomal miR-1247-3p released from high-metastatic HCC cells promotes tumor development by activating the β1-integrin-NF-κB signaling pathway and releasing pro-inflammatory cytokines, including IL-6 and IL-8.^205^ HCC cell-derived exosomal miR-210 can be transferred into ECs and enhances angiogenesis by targeting SMAD4 and STAT6.^206^ Similarly, miR-155 shuttled by exosomes from hypoxia-treated HCC cells is involved in tube formation of ECs and tumor angiogenesis.^207^ In addition, overexpression of exosomal miRNAs, including miR-224, miR-21, miR-92b, miR-93, miR-10b-5p, hsa-miRNA-1298, and miR-215-5p can be used as diagnostic markers for patients with HCC.^202,203,208–212^ On the contrary, levels of some exosomal miRNAs, including miR-9-3p, miR-125b, miR-638, miR-718, miR-101, miR-106b, miR-122, miR-195, and miR-744 are downregulated in HCC patients.^213–218^

LncRNAs shuttled by exosomes play key roles in regulating tumor cell proliferation, angiogenesis, invasion, and metastasis.^219^ Li et al. revealed that lncRNA FAL1 is highly expressed in serum exosomes from HCC patients and promotes HCC cell proliferation and metastasis through competitively binding to miR-1236.^220^ H19 is upregulated in exosomes from propofol-treated HCC cells and stimulates tumor cell proliferation, migration, and invasion through the miR-520a-3p/LIMK1 axis.^221^ Moreover, exosomal lncRNA H19 is significantly upregulated in CD90^+^ liver cancer cells and is implicated in promoting angiogenesis and regulating tumor microenvironment.^222^ In addition, lncRNA TUC339 is highly expressed in exosomes from HCC cells and it stimulates M2 macrophage polarization, thus enhancing tumor cell migration, invasion, and EMT.^223^ Similarly, HCC-derived exosomal DLX6-AS1 triggers M2 macrophage polarization by targeting the miR-15a-5p/CXCL17 axis.^71^ LincRNA VLDLR encapsulated in extracellular vesicles was highly expressed in HCC cells and promotes cellular stress responses.^224^ Sorafenib-treated HCC cell-derived exosomal lincRNA ROR is upregulated and suppresses death of recipient HCC cells by targeting the p53 signaling pathway.^225^ Ma et al. reported that expression of exosomal ASMTL-AS1 was highly correlated with the stage, metastasis, and prognosis in HCC.^226^ High expression level of exosomal lncRNA-ATB is significantly correlated with lower overall survival in HCC patients. Therefore, exosomal lncRNA-ATB is a promising prognostic biomarker for HCC.^208^ In addition, expression levels of LINC00161, LINC00635, lncRNA-RP11-583F2.2, lnc-FAM72D-3, lnc-EPC1-4, and lncRNA-HEIH in exosomes are high in HCC patients.^212,227–230^ On the contrary, SENP3-EIF4A1 and linc-FAM138B are downregulated in plasma exosomes in HCC patients. Exosomal SENP3-EIF4A1 can be transferred into HCC cells thus inhibiting tumor cell growth, and attenuate invasion and migration of HCC cells,^231^ while exosomal linc-FAM138B plays a role in repressing HCC growth by targeting miR-765.^232^

Exosomal circRNAs play a role in cellular processes of HCC, such as cell proliferation, angiogenesis, metastasis, and EMT. Huang et al. claimed that exosomal circRNA-100338 secreted by highly metastatic HCC cells is significantly upregulated and can be transferred to human umbilical vein endothelial cells (HUVECs). Exosomal circRNA-100338 can stimulate cell proliferation, angiogenesis, and vasculogenic mimicry formation of HUVECs and promote tumor metastasis.^233^ In addition, exosomal circRNA Cdr1as is highly expressed in HCC cells and can be transferred to surrounding cells. Overexpression of Cdr1as stimulates cell proliferation and migration by targeting the miR-1270/AFP axis.^234^ Circ-ZNF652 is highly expressed in exosomes from HCC cells and in serum of HCC patients. Exosomal circ-ZNF652 is implicated in stimulating cell proliferation, migration, invasion, and glycolysis by targeting miR-29a-3p/GUCD1 axis.^235^ Moreover, expression of circFBLIM1 is upregulated in HCC serum exosomes and promotes tumorigenesis and glycolysis by targeting miR-338.^236^ Exosomal circ-0004277 plays a role in inducing malignant phenotype of HCC by suppressing expression of ZO-1 and increasing tumor cell progression, migration, and EMT.^237^ In addition, the level of serum exosomal CircPTGR1 is upregulated in HCC patients and plays a role in facilitating tumor metastasis by targeting miR449a/MET pathway.^238^ Overexpression of exosomal has-circ-0039411 increases secretion of matrix metallopeptidase 2 (MMP2) by sponging miR-136-5p. High expression levels of exosomal has-circ-0039411 and MMP2 are correlated with tumor metastasis and low overall survival of HCC patients.^239^ HCC cell-derived exosomes exhibit a high expression level of circUHRF1. Exosomal circUHRF1 can trigger dysfunction of natural killer (NK) cells thus inducing immunosuppression in HCC patients.^240^ Expressions of exosomal circRNAs, such as circAKT3, has-circ-0004001, has-circ-0004123, has-circ-0075792, circ-0061395, and circTMEM45A are also highly upregulated in HCC patients.^241–243^ However, exosomal circ-0051443 is downregulated in plasma exosomes from HCC patients, which exerts a role in inducing cell apoptosis and inhibiting malignant behaviors.^244^ Wang et al. reported that level of exosomal has-circ-0074854 is also downregulated in HCC patients and plays a role in inhibiting tumor cell migration and invasion by repressing M2 macrophage polarization.^245^

#### Exosomal ncRNAs in CRC

CRC is a common cause of cancer-associated deaths worldwide. Approximately 1.2 million CRC cases are diagnosed and 600,000 people die with CRC every year.^246^ Previous studies report that exosomal ncRNAs play important roles in CRC.

Exosomal miRNAs exert vital effects on different progression of CRC.^247^ First, exosomal miR-183-5p promotes tumor development and induces cell proliferation, invasion, and tube formation of ECs.^248^ Second, exosomal miR-25-3p and miRNA-146a-5 are corelated with angiogenesis and tumorigenesis of CRC, respectively.^249,250^ Third, high expressed miR-17-5p in exosomes promotes CRC metastasis.^251^ Fourth, exosomal miR-210 plays a role in promoting EMT and anoikis resistance.^252^

Besides, several exosomal miRNAs modulate the metastasis of CRC. CRC-derived exosomal miR-934 is involved in inducing liver metastasis of CRC by mediating cellular communication between tumor-associated macrophages and CRC cells.^253^ In addition, miR-1255b-5p is highly expressed in hypoxic-treated exosomes from mouse CRC. Exosomal miR-1255b-5p inhibits EMT, CRC development, and liver metastasis by regulating expression of human telomerase reverse transcriptase (hTERT).^254^ Furthermore, high-metastatic CRC-derived exosomal miR-106b-3p induces tumor cell migration, invasion, EMT, and lung metastasis by targeting deleted in liver cancer-1 (DLC-1).^255^

Numerous studies have found that expression levels of serum exosomal miRNAs, including let-7a, miR-1229, miR-1246, miR-150, miR-21, miR-223, miR-23a, miR-301a, miR-17-5p, miR-92a-3p, miR-6803-5p, and miR-320d are significantly upregulated in primary CRC patients.^256–260^ In addition, some plasma exosomal miRNAs, such as miR-27a and miR-130a are upregulated in CRC and can serve as noninvasive biomarkers for CRC.^261^ On the contrary, low expression levels of exosomal miRNAs, including miR-874, miR-30a-5p, and miR-128-3p are highly correlated with tumor metastasis, differentiation, and advanced TNM stage.^262–264^

Moreover, exosomal lncRNAs play important roles in CRC. Exosomal RPPH1, MALAT1, NNT-AS1 promotes tumor progression, including cell proliferation, invasion, migration, and metastasis,^265,266^ while exosomal LINC02418 and H19 induces tumorigenesis and development.^267,268^ Exosomal HOTTIP is highly expressed in mitomycin-resistant CRC cells and promotes mitomycin resistance.^269^ Notably, low levels of exosomal HOTTIP are highly correlated with poor overall survival of CRC patients.^270^ Besides, studies report that exosomal lncRNAs, including GAS5, CRNDE-h, CRNDE-p, LINC02418, CCAT2, LNCV6-116109, LNCV6-98390, LNCV6-38772, LNCV-108266, LNCV6-84003, LNCV6-98602, FOXD2-AS1, NRIR, and XLOC-009459 are high expressed in CRC patients.^267,271–276^ In addition, serum exosomal lncRNA UCA1 is downregulated in CRC patients.^277^

Recent studies reported that exosomal circRNAs are involved in pathophysiology of CRC. Shang et al. determined that exosome-encapsulated circPACRGL from CRC patients stimulates tumor cell proliferation, migration, invasion, and metastasis, as well as neutrophil differentiation.^278^ Moreover, exosomal circFMN2 mediates cell proliferation and migration,^279^ while exosomal circIFT80 is implicated in promoting CRC development.^280^ Exosomal has-circ-0005963 and circ-133 regulate the process of CRC by targeting miR-122/M2 isoform of pyruvate kinase (PKM2) axis^281^ and miR-133a/GEF-H1/RhoA axis,^282^ respectively. Circulating exosomal hsa-circ-0004771 is significantly upregulated in CRC patients and is a novel potential diagnostic biomarker of CRC.^283^

#### Exosomal ncRNAs in GC

GC is a common cause of cancer-related death worldwide. In 2018, approximately 784,000 GC-related deaths were reported.^284^ Emerging evidence suggested that exosomal ncRNAs are involved in development of GC.

Cellular proliferation, migration, and invasion are closely associated with GC. Exosomal miR-1290 is upregulated in GC patients and stimulates proliferation, migration, and invasion of GC cells by downregulating expression of naked cuticle homolog 1 (NKD1).^285^ GC tissue-derived mesenchymal stem cells (GC-MSCs)-derived exosomal miR-221 play a role in tumor cell growth and migration.^286^ Exosomal miR-301a-3p promotes progression and metastasis of GC cells by regulating PHD3/HIF-1α.^287^ Exosomal miR-15b-3p promotes tumor cell proliferation, migration, and invasion by targeting DYNLT1/Caspase-3/Caspase-9 pathway.^288^ In addition, miR-34 packaged in exosomes from gastric CAFs shows low expression levels. Notably, overexpression of exosomal miRNA-34 can inhibit tumor cell proliferation, invasion, and motility.^289^

Besides, exosomal miRNAs take part in the progression of metastasis and angiogenesis of GC. MiR-let-7 is highly expressed in metastatic GC cell-derived exosomes. Exosomal miR-let-7 promotes tumorigenesis and metastasis by targeting RAS and HMGA2.^290^ Exosomal miR-21-5p promotes GC peritoneal metastasis by stimulating mesothelial-to-mesenchymal transition of peritoneal mesothelial cells (PMCs). Mechanistically, exosomal miR-21-5p induces metastasis by targeting SMAD7 and activating TGF-β/Smad signaling pathway.^291^ Wang et al. reported that exosomes released from GC cells exhibit a high level of miR-27a. Exosomal miR-27a induces CAFs and promotes tumor cell motility and metastasis by targeting CSRP2.^292^ Gastric CAFs-derived exosomal miR-139 suppresses gastric cancer metastasis and development by reducing matrix metalloproteinase 11 (MMP11).^293^ GC cell-derived exosomal miR-130a can be delivered into ECs to induce angiogenesis and tumor growth by regulating c-MYB.^294^ Studies report that exosomal miRNAs, such as miR-19b-3p, miR-106a-5p, miR-1246, miR-107, miR-196a-1, miR-106a, and miR-155-5p are all upregulated in GC patients.^295–300^

Exosomal lncRNAs regulate the progression of GC through diverse mechanisms. Overexpression of exosomal HOTAIR induces proliferation, migration, and invasion of GC cells by increasing KRAS.^301^ Exosomal LINC01559 promotes tumor progression by regulating miR-1343-3p/ phosphoglycerate kinase 1 (PGK1) and activating PI3K/AKT pathway.^302^ GC cells-derived exosomal lncRNA HEIH can be delivered into normal gastric cells and promote malignant transformation of GC by promoting expression of EZH2.^303^ Wang et al. reported that exosomal HOTTIP is implicated in cisplatin resistance in GC patients by targeting miR-218/HMGA1 axis.^304^ Numerous lines of evidence suggested that exosomal lncRNAs, such as UEGC1, HOTTIP, GC1, MIAT, H19, lnc-SLC2A12-10:1, CEBPA-AS1, ZFAS1 are highly expressed.^118,305–311^ On the contrary, exosomal lncRNAs, such as GNAQ-6:1 and PCSK2-2:1 show low expression levels in GC patients.^312,313^

Exosome-mediated circRNAs play important roles in GC. Xie et al. demonstrated that circSHKBP1 is highly expressed in exosomes from GC patients and the level of exosomal circSHKBP1 was reduced after gastrectomy. Exosomal circSHKBP1 promotes proliferation, migration, invasion, and angiogenesis of GC cells by targeting miR-582-3p/HUR/VEGF axis and through inhibition of HSP90 degradation.^314^ CiRS-133 encapsulated in exosomes secreted by GC cells can be transferred to preadipocytes. Exosomal ciRS-133 stimulates preadipocytes differentiation into brown-like cells by regulating miR-133/ PRDM16 pathway.^315^ Exosomal circNEK9 promotes proliferation, migration, invasion, and motility of recipient GC cells by modulating miR-409-3p/MAP7 axis.^316^ Moreover, circ-PVT1 is highly expressed in cisplatin-resistant GC cells-derived exosomes. Exosomal circ-PVT1 promotes cisplatin resistance in GC cells by inducing cell autophagy and invasion and by inhibiting apoptosis. Overexpression of exosomal circ-PVT1 induces low expression of miR-30a-5p and high expression of YAP1 in GC cells.^106^ Circ29 packaged in exosomes can be transferred from GC cells to ECs and promote proliferation, migration, and tube formation of ECs by targeting the miR-29a/VEGF pathway.^317^ In addition, circNHSL1 is highly expressed in exosomes released from GC cells. Exosomal circNHSL1 promotes migration, invasion, and glutaminolysis of GC cells by targeting miR-149-5p/YWHAZ axis.^318^ Overexpression of exosomal circ-0032821from oxaliplatin-resistant GC cells promotes proliferation, migration, and invasion of GC cells by modulating miR-515-5p/SOX9 pathway.^319^ Notably, exosomal has-circ-0065149 is downregulated in GC patients.^320^

#### Exosomal miRNAs in PCa

PCa is a heterogeneous disease.^321^ Approximately 160,000 PCa cases are diagnosed each year in the United States.^322^ Accumulating studies indicate that exosomes are implicated in PCa tumor development. Dysregulated exosomal ncRNAs are involved in tumor initiation and progression of PCa.^323^

Exosomal miRNAs are implicated in PCa. MiR-217 encapsulated in exosomes exerts roles in promoting tumor cell proliferation, invasion, and EMT.^324^ MiR-1246, a tumor inhibitor, was downregulated in PCa cell-derived exosomes. Overexpression of exosomal miR-1246 inhibits tumor cell proliferation, migration, and invasion and promoted cell apoptosis by suppressing EMT. Exosomal miR-205 released from human bone marrow mesenchymal stem cells (hBMSCs) promotes tumor cell apoptosis and inhibits proliferation, migration, and invasion of PCa cells by targeting RHPN2.^325^ Exosomal miR-26a exerts a vital role in mediating tumor growth and tumor cell metastasis.^326^ Tumor-associated macrophages enhances PCa progression through exosomal miR-95.^327^ MiR-183 encapsulated in exosomes is upregulated in PCa patients and promotes proliferation, migration, and invasion of tumor cell by downregulating expression of TPM1.^328^ Ye et al. reported that exosomal miR-141-3p promotes osteoblastic metastasis of PCa by modulating activity of osteoblasts.^329^ Huang et al. suggested that overexpression of plasma exosomal miR-1290 and -375 was significantly correlated with poor overall survival of PCa.^330^ Plasma exosomal miRNAs such as miR-1285, miR-622, miR-221, miR-145, and serum exosomal miRNAs such as miR-1274a, miR-1207-5p, miR-885-5p, miR-874, miR-766, miR-640, miR-636, miR-486-5p, miR-375, miR-346, and miR-141 are significantly upregulated in PCa patients and are promising biomarkers for diagnosis of PCa.^331,332^

Exosomal lncRNAs are implicated in diverse molecular processes involved in PCa progression. LncRNA MYU shuttled by exosomes is highly expressed in PCa patients and can be delivered into adjacent cells. Notably, exosomal lncRNA MYU induces cell proliferation and migration by competitively binding miR-184 and promoting expression of c-Myc.^333^ PCa-derived exosomal SChLAP1 is highly expressed and significantly correlated with the level of prostate specific antigen (PSA) and tumor cell invasion.^334^ Ozgur et al. demonstrated that exosomal H19 is involved in PCa by regulating androgen receptor pathway.^335^

CircRNAs highly expressed in exosomes can be transferred from tissues into various body fluids and are potential diagnostic biomarkers for PCa.^336^ Has-circ-0044516 shuttled by exosomes induces tumor cell metastasis and inhibits tumor cell apoptosis by sponging miR-29a-3p.^337^ Exosomal circ-XIAP is upregulated in docetaxel-resistance PCa cells and promotes docetaxel resistance by targeting miR-1182/TPD52 axis.^338^

In general, exosomal ncRNAs are involved in pathological cellular processes including cell proliferation, migration, invasion, metastasis, angiogenesis, and EMT associated with diverse cancers. Accumulating evidence over the last decade has further revealed that exosomal ncRNAs can participate in multiple processes contributing to cancer development, diagnosis biomarkers, and therapeutic effects, showing the dual characteristics of promoting and suppressing cancer. In this part, we mainly discuss the roles of exosomal miRNAs, lncRNAs, and circRNAs in lung cancer, BC, HCC, CRC, GC, and PCa. Notably, studies have explored roles of exosomal ncRNAs in other cancers, including esophageal cancer, pancreatic cancer, ovarian cancer, and leukemia.^339^

### The roles of exosomal ncRNAs in metabolic diseases

**Fig. 6:**
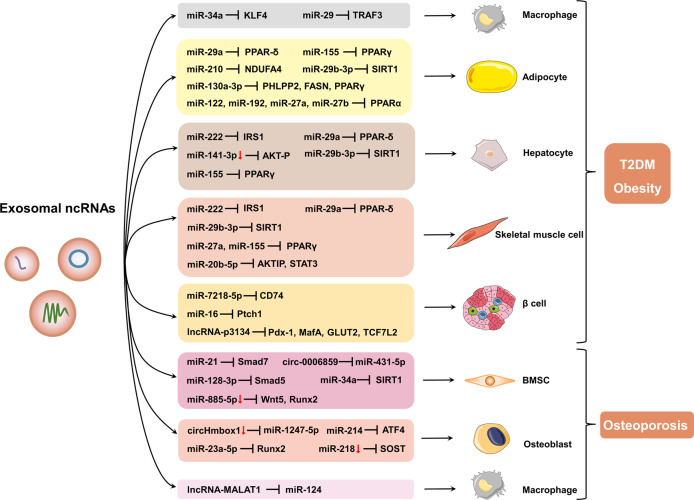
The role of exosomal ncRNA in the pathological process of metabolic diseases. Exosomes secreted by different tissues can be released into the circulation and transported to other organs, where they are internalized by recipient cells, mediating metabolic regulation. This figure was created with the aid of Servier Medical Art (https://smart.servier.com/)

Previous studies report that exosomal ncRNAs play important roles in remote tissues. NcRNAs-encapsulated exosomes are implicated in various processes that involved in development of metabolic diseases, such as T2DM, obesity, and osteoporosis (Fig. 6).

#### Exosomal ncRNAs in T2DM

T2DM is a prevalent chronic disease that causes cardiovascular, renal, retinal, and neurological complications, and is a major cause of death and disability worldwide.^340,341^ T2DM is the most common clinical type of diabetes accounting for over 90% of diabetes cases, which is characterized by relative insulin deficiency as a result of progressive inadequate insulin secretion and varying degrees of IR in peripheral tissues, such as adipose tissues, skeletal muscle, and liver tissues.^9,342^ Aging, obesity, and other cardiovascular risk factors promote the development of T2DM.^340^ However, the roles of these factors in promoting the pathological process in T2DM has not been fully elucidated. Recent studies on exosomes and their ncRNA cargoes have been conducted to explore the underlying mechanisms.

MiRNAs are the most explored ncRNAs, and several exosomal miRNAs are closely linked to T2DM. Adipose tissue is an endocrine organ that functionally regulates systematic energy homeostasis by releasing various endocrine factors which modulate glucose and lipid metabolism.^9,343^ In addition, adipose tissue is a major source of circulating exosomal miRNAs, which modulate glucose tolerance and the expression of fibroblast growth factor 21 (FGF21) in the liver.^344^ MiRNAs profiles in adipocyte-derived exosomes vary under different physiopathological conditions of adipose tissue.^345^ Adipocyte-derived exosomal miRNAs, such as miR-34a,^346^ miR-222,^347^ miR-27a,^348^ and miR-802-5p^85^ can promote IR via regulating Krüppel-like factor 4 (KLF4), insulin receptor substrate 1 (IRS1), peroxisome proliferator-activated receptor γ (PPARγ), and HSP60. Hepatocyte uptake of exosomes from adipocytes in obese mice containing less miR-141-3p than that in healthy mice, and the reduced absorption of miR-141-3p resulted in reduced glucose uptake in hepatocytes.^349^ A recent study reported that exosomal miR-130b-3p from adipocytes isolated from epididymal fat of HFD mice or incubated with high glucose/high lipid level aggravates myocardial ischemia/reperfusion injury in nondiabetic mice, by inhibiting various antiapoptotic and cardioprotective signaling, mainly AMP-activated protein kinase (AMPK).^350^ In addition to adipocytes, macrophages can release exosomes containing miRNAs that regulate glucose metabolism. Adipose tissue macrophage (ATM)-derived exosomal miR-155 in obese mice promotes glucose intolerance and IR by targeting PPARγ.^351^ Similarly, ATM-derived exosomal miR-29a can be transported into adipocytes, myocytes, and hepatocytes, thus inducing IR in vitro and in vivo through PPAR-δ.^352^ MiR-690 in exosomes derived from M2-polarized macrophages can alleviate glucose tolerance and IR in obese mice by targeting Nadk, a gene encoding NAD^+^ kinase.^353^ Exosomal miR-210 released from high glucose (HG)-induced ATM promotes the development of diabetic obesity in mice by inhibiting NADH dehydrogenase ubiquinone 1 alpha subcomplex 4 (NDUFA4) in adipocytes, leading to reduced glucose uptake and mitochondrial complex IV (CIV) activity.^354^ Exosomal miR-21-5p derived from HG-stimulated macrophages induces inflammation, ROS production, and podocyte injury in diabetic nephropathy (DN) mice by modulating A20.^79^

Liver-derived exosomal miR-130a-3p ameliorates glucose intolerance and IR by repressing pleckstrin homology domain leucine-rich repeat protein phosphatases 2 (PHLPP2) thus triggering AKT/Akt substrate of the 160 kDa (AS160)/glucose transporter 4 (GLUT4) axis in adipocytes.^355^ Expression level of hepatocyte-released exosomal miR-7218-5p in HFD mice is lower compared with that in normal diet mice. MiR-7218-5p mimic transfection decreases islet β cells proliferation by regulating CD74.^356^

Furthermore, skeletal muscle-derived exosomal miRNAs play important roles in T2DM, although only a few studies have explored these roles. Jalabert and colleagues found that exosomes extracted from quadriceps muscle in mice fed with high palmitate diet promoted proliferation of β cells and isolated islets. MiR-16 is upregulated in these exosomes and can modulate Ptch1 associated with pancreas development.^357^

It has been reported that islet β cells can secretes different miRNAs. When encapsulated by exosomes, the miRNAs can be transported to functionally act on different recipient cells.^358^ Specifically, it has been shown that β cell-derived exosomal miR-29s attenuate hepatic insulin sensitivity and regulate glucose homeostasis as a result of high levels of free fatty acids.^359^ Another study shows that exosomal miR-29 induces recruitment and activation of monocytes and macrophages as well as consequent inflammation in HFD mice. This promotes progression of diabetes by decreasing tumor receptor-associated factor 3 (TRAF3).^42^ The abundance of miR-26a is decreased in serum exosomes of overweight human and obese mice and is negatively correlated with clinical characteristics of T2DM. Several experiments have proven that exosomal miR-26a from β cells can ameliorate peripheral IR induced by obesity. Moreover, upregulation of miR-26a in β cells reduces glucose-stimulated insulin secretion (GSIS) by inhibiting actin cytoskeleton remodeling and thus prevents obesity-induced islet hyperplasia.^360^ Exosomal miR-15a released from pancreatic β-cells can travel through the circulation and be absorbed by Müller cells. This triggers oxidative stress and causes retinal injury and apoptotic cell death under T2DM conditions.^361^ Additionally, β cell-derived exosomal miR-127 enhances the migration and ability of islet ECs to form tubes.^362^

Bone marrow-derived MSC (BMSC)-derived exosomal miR-29b-3p in aged mice can be absorbed by adipocytes, myocytes, and hepatocytes, which subsequently decreases insulin sensitivity both in vivo and in vitro via targeting SIRT1. Intriguingly, downregulation of nanocomplex-mediated miR-29b-3p in exosomes from BMSCs attenuates IR in aged mice.^40^

Circulating exosomal miR-20b-5p attenuates insulin-stimulated glycogen accumulation through regulating AKT-interacting protein (AKTIP) and STAT3.^363^ MiRNA profiles in plasma exosomes were altered in obese mice, including increased miR-122, miR-192, miR-27a-3p, and miR-27b-3p. By targeting PPARα, the treatment of lean mice with exosomes containing obesity-related miRNAs induces glucose intolerance and IR.^78^ Additionally, altered miRNAs encapsulated in circulating exosomes correlates with the adiponectin pathway in T2DM patients.^364^

In addition to miRNAs, exosomal lncRNAs are also emerging as significant factors in T2DM. Expression of serum exosomal lncRNA-p3134 was elevated in T2DM patients.^365^ This lncRNA has been revealed to be interrelated with levels of fasting blood glucose and homeostasis model assessment β-cell function (HOMA-β). Overexpression of lncRNA-p3134 can promote GSIS and reduce apoptosis in β cells, which provides a novel mechanism of glucose homeostasis regulation by lncRNAs.^365^ The circulating level of exosomal lncRNA-MALAT1 significantly decreases in patients with T2DM.^366^

Exosomal lncRNAs also exert protective effect. Li and coworkers have shown that exosomal lncRNA H19 from MSCs can induce fibroblast proliferation and migration, as well as inhibit apoptosis and inflammation via abrogating miR-152-3p-mediated PTEN repression and hence facilitates diabetic wound healing.^367^ Exosomes-mimetic nanovesicles carrying a high level of H19 can serve as a nano-drug delivery system to neutralize the inhibitory effect of hyperglycemia on regeneration and speed up chronic wound healing.^368^ MSC-released exosomal lncRNA SNHG7 inhibits HG-stimulated endothelial-mesenchymal transition and tube formation of human retinal microvascular ECs by targeting miR-34a-5p/XBP1 axis. This provides a promising therapeutic method for diabetic retinopathy.^369^

Emerging evidence has unveiled the important role of exosomal circRNAs in T2DM complications. Our recent study has revealed that HG-stimulated ECs release exosomes containing circRNA-0077930 to vascular smooth muscle cells (VSMCs) causing VSMC senescence,^370^ which may lead to diabetic vascular complications. Further, circRNAs in serum exosomes were different among patients with diabetic foot ulcer (DFU), non-DFU diabetes, and healthy cases.^371^ Among these modified circRNAs, the abundance of exosomal has-circ-0000907 and has-circ-0057362 were significantly increased in early DFU. Further experiments showed their role as promising biomarkers in the early diagnosis of DFU.^371^ It has been reported that circRNA cPWWP2A can be transported from retinal pericytes to ECs by exosomes in a paracrine manner. Further, high expression of cPWWP2A attenuates diabetes-induced retinal vascular dysfunction in vivo.^372^ Besides, a recent study has verified that serum exosomal circRNA DLGAP4 was elevated in patients with DN and rat models as compared with T2DM individuals without DN and normal rats. Furthermore, exosomal circRNA DLGAP4 have been shown to induce mesangial cell proliferation and fibrosis, as well as exacerbating DN in vivo by sponging miR-143 to motivate Erb-b2 receptor tyrosine kinase 3 (ERBB3)/NF-κB/matrix metalloproteinase-2 (MMP-2) axis.^373^

Collectively, exosomal miRNAs from a variety of sources, including adipose tissue, liver, skeletal muscle, islet β cells, BMSC, are all involved in the pathological process of T2DM. Exosomal lncRNAs not only act as modulators (lncRNA-p3134 and MALAT1), but also have therapeutic effects on T2DM and its complications (H19 and SNHG7). Exosomal circRNAs mainly play vital roles in T2DM complications, such as DFU, DN, and retinal vascular disorders.

#### Exosomal ncRNAs in obesity

Obesity is defined as abnormal or excessive accumulation of body fat that presents a health risk to an individual. According to World Health Organization, obesity is diagnosed with a body mass index (BMI) greater than 30 kg/m.^2,374^ Obesity has grown into an epidemic with approximately 604 million adults affected worldwide in 2015.^375^ This disorder has caused enormous social burden due to multiple comorbidities such as diabetes, hyperlipidemia, cardiovascular diseases, and diverse cancers.^376^ Understanding the pathogenesis of obesity contributes to effective management of this harmful disorder.

Emerging evidence reveals that exosomes, as a mediator that intercellularly transports ncRNAs, play a pivotal role in the development of obesity and associated metabolic disorders. Santamaria-Martos and coworkers detected several plasma exosomal miRNAs candidates associated with BMI (e.g., let-7b, miR-146a), dyslipidemia (miR-29c) and fasting insulin (e.g., miR-222/223, miR-26b) in obese and non-obese women.^377^ Moreover, it has been reported that exosomal lncRNA-H19 expression is correlated with waist circumference.^366^ Elsewhere, exosomal miR181b‑5p and miR219‑5p of immune cell origin that are induced by elafin (an anti-inflammatory protein) promote leptin expression in adipocytes to reduce food consumption, obesity, and hyperglycemia in HFD male mice.^378^ It has been reported that aerobic exercise regulates serum exosomal miRNAs in obese mice. It decreases the levels of miR-122, miR-192, and miR-22, which are associated with improved adipogenesis, insulin sensitivity, and hepatic steatosis.^379^ Similarly, exosomal miRNAs profiles are modified in obese patients after bariatric surgery.^380,381^

Obesity is recognized as a state with chronic low-grade inflammation.^59^ Adipose tissue is an active endocrine organ that releases a variety of cytokines, such as adiponectin, interleukins, and TNF-α. It also secretes other pro-inflammatory biomolecules including miRNAs, which leads to metabolic dysfunction, particularly IR.^126^ MiRNAs contained in exosomes have been identified as important mediators of the inflammation caused by obesity. Some exosomal miRNAs are identified to be differentially expressed in visceral adipose of obese subjects compared to lean subjects. Besides, these miRNAs might target genes associated with obesity-induced inflammation, such as transforming growth factor-β (TGF-β) and Wingless and int-1 (Wnt)/β-catenin signaling.^382,383^ Moreover, circulating exosomal miRNAs from obese mice have also been found to trigger adipose tissue inflammation in lean mice.^78^ Elsewhere, exosomal miR-34a from obese mice have been found to promote inflammation by regulating the M1-to-M2 macrophage ratio.^346^ Additionally, exosomal miR-690 from M2-polarized macrophages regulates inflammation in obese mice.^353^

There is direct or indirect association of obesity with IR disorder.^384^ It has been reported that exosomal miRNAs profiles are modified by dysregulation of glucose metabolism in obese patients.^385^ On the other hand, obesity can contribute to IR through transporting exosomal miRNAs to target cells and tissues, which participates in a variety of pathophysiological processes including inflammation response and insulin signaling pathway.^76,384^ As previously stated, exosomal miRNAs from adipose tissue, particularly miR-155, miR-34a, miR-222, miR-27a, miR-29a, miR-210, and miR-141-3p, are crucial regulators in the physiopathology of obesity-induced IR.^346–349,351,352,354^ In addition, miR-192, miR-122, miR-27a-3p, and miR-27b-3p are upregulated in plasma exosomes in obese mice. Injection of exosomes transfected with these increased miRNAs contributes to central obesity in lean mice by targeting PPARα.^78^ On the contrary, miR-26a from β cells and miR-690 from M2-polarized macrophages which are contained in exosomes can ameliorate IR induced by obesity.^353,360^

In summary, exosomal ncRNAs participate in regulation of obesity and are related to BMI, dyslipidemia, and waist circumference. In addition, they are considered to be crucial mediators of inflammation caused by obesity and IR disorder.

#### Exosomal ncRNAs in osteoporosis

Osteoporosis is characterized by loss of bone mass, degradation of bone microstructure. The disease contributes to increased bone fragility and risk of fracture.^386,387^ As the global population ages, the incidence of osteoporotic fractures is increasing with consequent enormous economic costs, reduced quality of life and lifespan.^388,389^ The maintenance of bone mass relies on the strictly coordinated balance between bone formation and bone resorption, and the main cells involved are osteoblasts and osteoclasts. Osteoporosis is accompanied by an increase in bone resorption and a decrease in bone formation. Recent studies have demonstrated that some exosomal ncRNAs play a pivotal part in the modulation of osteogenesis and bone resorption.^390,391^

Exosomal miRNAs derived from MSCs exert a potential regulatory effect on osteogenesis.^392^ During osteogenic differentiation in human BMSCs, the miRNAs profile contained in exosomes is changed during osteogenic differentiation in human BMSCs. Among the effected changes, miR-885-5p is expression of miR-885-5p is lowered and proven to serve as a negative regulator of BMSCs osteogenic differentiation by suppressing Wnt5 and runt-related transcription factor 2 (Runx2).^393^ According to Jiang et al., miR-21-bearing exosomes in osteoporosis patients repress osteogenesis by targeting small mothers against decapentaplegic homolog 7 (Smad7).^394^ Further, a study by Xu et al. reported that exosomal miR-128-3p derived from MSCs in aged rats inhibits osteogenesis and fracture healing through dampening Smad5.^390^

Emerging evidence indicates that osteoclast-derived exosomes can transfer miRNAs to osteoblast that regulate bone formation. Elsewhere, Sun et al. revealed that osteoclast-derived exosomes shuttling miR-214 represses the osteoblasts activity.^395^ Further, Li et al. demonstrated that exosomal miR-214-3p could reduce osteoblast activity in vitro and impair bone formation in vivo. On the contrary, inhibition of miR-214-3p in osteoclast was reported to facilitate bone formation in aging ovariectomized mice.^396^ Moreover, Yang and colleagues showed that osteoclast-released exosomes enriched with miR-23a-5p inhibits osteogenic differentiation via repressing Runx2.^397^

Exosomes can mediate muscle-bone crosstalk through transporting their miRNAs cargoes. Fulzele et al. proved that circulating muscle-derived exosomal miR-34a increases with age in mice. High expression of miR-34a in the exosomes from myoblasts impaired BMSC viability and promoted cellular senescence, as well as decreased SIRT1 expression in BMSCs.^398^ Other studies have also reported that Sirt1 promotes differentiation of osteoblast.^399,400^ These findings suggests that there is a potential pattern of inter-organ crosstalk which causes physiopathology of bone with age. Myostatin, a muscular-secreted myokine, has been revealed to exert modulating effects on bone mass. Notably, it was found that downregulation of exosomal miR-218 derived from osteocytes could mediate inhibition of osteoblastic differentiation that is induced by myostatin through repression of sclerostin (SOST).^401^

Exosomal lncRNAs also exert important roles in osteoporosis. Teng et al. detected the levels of circulating exosomal lncRNAs in osteoporotic patients compared with normal subjects and identified 393 differentially expressed lncRNAs.^402^ Further bioinformatics analysis suggested that these lncRNAs may be associated with several osteoporosis pathways.^402^ Elsewhere, Cui et al. reported that lncRNA MALAT1 encapsulated by exosomes from endothelial progenitor cells (EPCs) could promote osteoclastogenesis of bone marrow-derived macrophages.^403^ This effect is achieved by MALAT1 serving as a miR-124 sponge to upregulate integrin subunit β 1 (ITGB1), which presents a pivotal role in osteoclastogenesis.^403^

Recently, exosomal circRNAs are recognized as a novel player in the pathophysiology of osteoporosis. CircRNAs in exosomes are differentially expressed in patients with osteoporosis. Among these circRNAs, exosomal hsa-circ-0006859 were upregulated and could differentiate individuals with osteopenia or osteoporosis from healthy individuals with high sensitivity and specificity. Mechanistically, hsa-circ-0006859 represses osteoblastic differentiation and induced adipogenic differentiation of human BMSCs through sponging miR-431-5p to promote ROCK1 expression.^404^ Exosomes containing a low concentration of circHmbox1 derived from TNF-α-treated osteoclasts decrease osteoblasts differentiation mainly by targeting miR-1247-5p. Overexpression of circHmbox1 remarkably mitigates the osteoporotic phenotypes in ovariectomized mice, which might function as a promising treatment strategy for postmenopausal osteoporosis.^405^

In a brief summary, exosomal miRNAs exert regulatory effects on osteogenesis, bone formation, and muscle-bone crosstalk. Exosomal lncRNAs are associated with a variety of osteoporosis pathways, such as MALAT1, which promotes osteoclastogenesis. Exosomal circRNAs are emerging as new regulators in the pathophysiology of osteoporosis. For instance, exosomal hsa-circ-0006859 is a highly sensitive and specific marker for patients with osteoporosis, while exosomal circHmbox1 exhibits a potential therapeutic effect for postmenopausal osteoporosis.

### The roles of exosomal ncRNAs in cardiovascular diseases

Despite the continuous progress in the treatment of cardiological ailments, cardiovascular diseases, such as hypertension, atherosclerosis (AS), acute myocardial infarction (AMI), heart failure (HF), and atrial fibrillation (AF), are still the leading cause of the morbidity and mortality worldwide.^406,407^ Recently, accumulating exosomal ncRNAs has been identified to be involved in the pathogenesis of cardiovascular diseases, which provides a new insight into the mechanisms and therapeutic targets for the diagnosis and treatment of these diseases.^408–413^ Moreover, exosomal ncRNAs serve as emerging regulators in dyslipidemia, thereby leading to an increase in risk of atherosclerotic cardiovascular diseases.^414^ For example, miR-26a is downregulated in circulating exosomes of overweight humans and obese mice, whereas, upregulation of miR-26a in mice reduces the abundance of plasma cholesterol, low-density lipoprotein (LDL) and HDL, hepatic triglyceride, as well as lipid droplets in adipose tissue.^360^ This study summarizes the current evidence about the roles of exosomal ncRNAs in cardiovascular diseases.

#### Exosomal ncRNAs in hypertension

Hypertension is an important risk factor for total mortality and cardiovascular disease, such as stroke, myocardial infarction, coronary heart disease, and HF.^415–417^ Emerging evidence indicates that diverse exosomal ncRNAs were implicated in the development of hypertension through modulating multiple cellular and molecular events, including renin-angiotensin-aldosterone system (RAAS), endothelial dysfunction, angiogenesis, VSMCs proliferation, vascular remodeling, inflammation, and oxidative stress.^418–420^

Current evidence reveals that exosomal miRNAs from different sources can modulate the initiation and progression of hypertension. Next-generation sequencing was used to detect the exosomal miRNA expression profile in spontaneously hypertensive rats (SHRs) and normotensive Wistar-Kyoto rats (WKYs). Liu et al. found that 23 exosomal miRNAs were significantly upregulated and 4 exosomal miRNAs were downregulated in SHRs compared to WKYs. The levels of exosomal miR-17-5p and miR-425-5p were markedly elevated in SHRs plasma and tightly associated with inflammation and blood pressure homeostasis, respectively.^421^ Besides, exosomal miR-155-5p derived from aortic adventitial fibroblast was downregulated in SHRs compared to WKYs. Low expression of exosomal miR-155-5p enhanced the expression of vascular angiotensin-converting enzyme and angiotensin II (Ang II) and promoted VSMCs proliferation, vascular remodeling, and hypertension.^422^

Macrophages-derived exosomal miRNAs play crucial roles in hypertension. It has been reported that THP-1 cells-derived exosomal miR-27a impaired vasodilation and increased rat blood pressure through inhibiting the expression of Mas receptor in ECs and endothelial nitric oxide synthase (eNOS) phosphorylation in mesenteric arteries.^423^ Additionally, miR-17 was decreased in Ang II-treated THP-1-derived exosomes and promoted ECs inflammation through increasing the expression of intercellular adhesion molecule-1 (ICAM-1) and plasminogen activator inhibitor-1 (PAI-1).^424^ The transfer of exosomal miR-106b-5p from macrophages to renal juxtaglomerular cell stimulated inflammation-induced hypertension by inhibiting transcription factors E2f1 and Pde3b.^425^

#### Exosomal ncRNAs in AS

AS is a chronic immune-inflammatory and age-related disorder which is characterized by lipid-rich plaques accumulated in the arterial wall.^426^ It is one of the main causes of cardiovascular diseases that leads to severe clinical outcomes like myocardial infarction and stroke.^427–429^ Accumulating evidence shows that various exosomal ncRNAs play an important regulatory role in the pathophysiological process of atherosclerosis. They were involved in the occurrence and development of atherosclerosis through regulating vascular inflammation, lipid metabolism, and cell survival.^430–432^

Several studies have demonstrated that exosomal miRNAs released by various types of cells are detected in circulation and are involved in the regulation of pathogenic AS.^410,433^ It has been reported that the levels of exosomal miR-223, miR-339 and miR-21 derived from thrombin-activated platelet were significantly upregulated.^434,435^ They could be transferred into VSMCs and inhibited the VSMCs proliferation stimulated by platelet derived growth factor.^434^ Besides, Li et al. also demonstrated that miR-223 inhibited TNF-α-stimulated endothelial cells (ECs) inflammation by decreasing the expression of ICAM-1.^435^ These findings indicate that exosomal miR-223 may play a protective role in AS through inhibiting the vascular inflammatory response.

The communication between exosomal miRNAs and ECs plays a vital role in the pathogenesis of AS.^436^ Exosomal miR-92a, upregulated by the combination of low shear stress and oxidized LDL in atherosclerotic mice model, promoted endothelial inflammation and atherosclerotic plaque formation.^437^ Besides, Xing et al. reported that exosomal miR-342-5p released by adipose-derived MSCs exerted an anti-atherosclerotic effect by promoting H_2_O_2_-induced ECs apoptosis and protecting against ECs injury.^438^ On the contrary, miR-155 derived from VSMCs destroyed the tight junction and integrity of ECs, which led to the ECs injury and might promote AS progress.^430^

The dysfunction of VSMCs mediated by macrophages-derived exosomal miRNAs also plays an important role in AS.^432,439^ A study by Zhu et al. reported that the expression of exosomal miR-21-3p was increased in nicotine-stimulated macrophages and could be assimilated by neighboring VSMCs.^432^ This effect resulted in an increase in capacities of VSMCs proliferation and migration and thus accelerated the development of AS. Further, it was found that exosomal miR-106a-3p derived from oxidized LDL-incubated macrophages can promote VSMCs proliferation and inhibit their apoptosis, which may protect individuals against AS.^439^

Macrophages are the main types of cells responsible for the inflammation reaction in AS. The role of macrophages is regulated by exosomal miRNAs which has attracted a lot of attention. It has been found that miR-let7 family is highly enriched with exosomes released by MSCs and inhibited the macrophages infiltration via IGF2BP1 pathway.^67^ Besides, it also promotes the polarization of M2 macrophages via HMGA2/NF-κB pathway, which ameliorates the progression of AS.^67^

In addition to miRNAs, it has been demonstrated that the expression of lncRNA GAS5 significantly increases in exosomes collected from patients or animals with AS.^440^ Further in vitro study revealed that lncRNA GAS5 promoted the apoptosis of macrophages and ECs induced by oxidized LDL, which may lead to the aggravation of AS.^440^ Besides, the expression of MALAT1, a lncRNA involved in tumorigenesis, was downregulated in oxidized LDL-induced ECs. Overexpression of exosomal MALAT1 derived from ECs increases the expression of MALAT1 in dendritic cells and inhibits the production of ROS and maturation of dendritic cells through activating NRF2 signaling pathway.^441^ This indicates that downregulation of exosomal MALAT1 derived from oxidized LDL-induced ECs may accelerate the development of AS through inducing dendritic cells maturation. Intriguingly, another study found an increase in the expression of ECs-derived exosomal MALAT1, which promotes the M2 macrophage polarization and may protect individuals against AS.^442^ However, another study found that the increase in expression of exosomal MALAT1 derived from ECs promotes the formation of neutrophil extracellular traps and accelerates the progression of AS.^443^

A few studies have found an association between exosomal circRNAs and the pathogenesis of AS.^444,445^ Wen et al. suggested that the expression of exosomal circRNA‑0006896 in circulation is positively associated with the levels of TG, LDL, and C-reactive protein (CRP) in patients with unstable plaque atherosclerosis.^445^ The study also reported that the elevated circulating exosomal circRNA‑0006896 leads to high expression of circRNA‑0006896 in ECs, which results in an increases in capacities of proliferation and migration in ECs. This effect is accompanied with the upregulation of DNMT1 and phosphorylation of STAT3, which may provide protection against the vulnerable plaque formation.^445^

#### Exosomal ncRNAs in AMI

AMI is characterized by the loss of cardiomyocyte and the necrosis of myocardial. It results from acute obstruction of the coronary artery and can leads to severe clinical consequences.^446^ Recent studies have revealed that exosomal ncRNAs play a vital role in AMI and may become effective biomarkers for diagnosis and treatment of AMI.^447–449^

During the pathogenesis of AMI, a wide range of exosomal miRNAs such as miR-125b, miR-499, miR-133, miR-22, miR-21, and miR-301 are found to be upregulated.^450–454^ Recent evidence has indicated that exosomal miRNAs are involved in the regulation of occurrence and development of AMI. This could be through regulation of pathophysiology pathways such as apoptosis, autophagy, inflammation, and angiogenesis.^447,455–457^

It has been demonstrated that miR-125b, miR-25-3p, miR-144, miR-126, and miR-146a encapsulated in exosomes from myocardial cells are enriched in expression and exert an anti-atherosclerotic role partly by inhibiting myocardial apoptosis and facilitating ischemic cardiac repair.^450,458–461^ Besides, exosomal miR-25-3p and miR-146a derived from myocardial cells were also found to restrain the inflammation response, whereas exosomal miR-301 inhibits myocardial autophagy.^454,458,461^ On the contrary, it was reported that circulating levels of exosomal miR-499 and miR-133a derived from cardiomyocytes are elevated in patients with acute coronary syndromes than those with stable coronary artery disease or without coronary artery disease.^451^ This implies that exosomal miR-499 and miR-133a may promote the progression of AMI.

In addition, exosomal miRNAs released by other types of cells other than cardiomyocytes also plays an important role in the pathogenesis of AMI. According to Feng et al. the expression of miR-22 in exosomes from MSCs is upregulated under ischemic condition.^452^ It is also revealed that exosomal miR-22 could be internalized by cardiomyocytes and promote the protection of cardiomyocytes against apoptosis via targeting methyl-CpG-binding protein 2 in vitro. Further in vivo experiments shows that delivery of exosomal miR-22 remarkably reduces cardiac fibrosis thus indicating their significant benefit in treating myocardial fibrosis after AMI.^452^ Besides, Wang et al. reported that enhanced expression of exosomal miR-21 derived from MSCs have a cardiac protective role through inhibiting apoptosis and promoting angiogenesis of ECs.^453^

Emerging evidence demonstrates an involvement of exosomal lncRNAs in AMI.^448,449,462,463^ According to Shyu et al. the expression of exosomal MALAT1 derived from hyperbaric oxygen-induced cardiomyocytes is significantly increased after AMI.^448^ The increase in MALAT1 then suppresses the expression of miR-92a and enhances the neovascularization. Huang et al. found an elevation in exosomal lncRNA H19 expression released by MSCs after treatment with atorvastatin. This led to an improvement of cardiac function recovery, a reduction of infarct size and cardiomyocyte apoptosis.^449^ Further, Wang et al. reported that the expression of exosomal lncRNA AK139128 from cardiomyocytes under hypoxic condition increases and this promotes the apoptosis but inhibits the proliferation, migration and invasion of cardiac fibroblasts.^463^

#### Exosomal ncRNAs in HF

HF results from dysfunction of myocardial systole or diastole. It is referred to as the decrease in cardiac blood output and the insufficient blood flow in the pulmonary or systemic circulation.^464^ The main mechanisms involved in HF include myocardial inflammation, autophagy, apoptosis, and remodeling. Recent studies have revealed that exosomal ncRNAs are involved in the regulation of pathogenesis of HF.^462,463,465^

During the pathogenesis of HF, a number of exosomal miRNAs are reported to be altered in the serum levels. It has been reported that exosomal miR-425 and miR-744 are lowered in the circulation of patients with HF.^466^ The downregulation of exosomal miR-425 and miR-744 are also reported in angiotensin II-treated cardiac fibroblasts and is positively related to the expression of collagen 1 and α-SMA, which leads to cardiac fibroblast fibrosis. Further in vitro experiments have revealed that exosomal miR-425 and miR-744 protect against myocardial remodeling by inhibiting angiotensin-II-induced collagen formation and fibrogenesis by targeting TGFβ1.^466^

#### Exosomal ncRNAs in AF

AF is a type of cardiac arrhythmia and is correlated with the structural and electrical remodeling.^467,468^ Recently, some studies have revealed that ncRNAs, especially miRNAs play an important role in pathological process of AF.^469–471^ According to Liu et al. there is low expression of miR-320d in AF cardiomyocytes, accompanied with an increase in apoptosis and impaired cell viability in cardiomyocytes.^469^ However, transfecting with exosomal miR-320d derived from adipose tissue-derived mesenchymal stem cells significantly reverses the effects of AF on cardiomyocytes, which is dependent on inhibition of STAT3. Besides, Wang et al. reported that the expression of exosomal miR-107 derived from patients with AF was significantly higher than the expression from patients without AF.^470^ Incubation of ECs with AF-derived exosomes or miR-107 mimics significantly inhibits cell viability and migration, while enhancing cell apoptosis by regulating miR-107/USP14 pathway. Furthermore, Li et al. demonstrated that miR-21-3p loaded in exosomes derived from myofibroblasts contributes to an increase in vulnerability of AF through upregulation of the expression of L-type calcium channel Cav1.2.^471^

In summary, exosomal ncRNAs are identified as key regulators in diverse cardiovascular diseases such as hypertension, AS, AMI, HF, and AF. Exosomal miRNAs are relatively well studied in cardiovascular diseases, which exert functions by inhibiting vascular inflammatory response, modulating cellular pathophysiology pathways, and so on. Exosomal lncRNAs (GAS5, MALAT1, H19, AK139128) are associated with AS and AMI. However, only a small number of exosomal circRNAs have been determined to be involved in the pathogenesis of AS.

### The roles of exosomal ncRNA in neurodegenerative diseases

The incidence of neurodegenerative diseases, including Alzheimer’s disease (AD) and Parkinson’s disease (PD) increases with age. Deviation of ncRNA levels in neurodegenerative diseases has been widely reported.^472–478^ Exosomes are demonstrated to carry ncRNAs and establish cell-to-cell communication in neurons. They are involved in the pathogenesis and progression of neurodegenerative diseases as mediators.

#### Exosomal ncRNA in AD

AD is the most common cause of dementia in elderly people. The pathological features of AD are extracellular Aβ deposition, intracellular neurofibrillary tangles, and neuronal loss.^479^ Exosomal ncRNAs have been shown to regulate the expression and function of amyloid precursor proteins (APP) and tau proteins.^480^

Growing evidence shows that exosomal miRNAs play an important role in AD.^481,482^ MiRNA expression profile alters in the brains of patients with AD.^483^ APP, β-site amyloid precursor protein-cleaving enzyme 1 (BACE1) and microtubule-associated protein tau (MAPT) are pathologically related proteins of AD.^484,485^ Neuropathological changes in AD are the consequence of diverse cellular processes, such as alterations of AD relevant proteins and oxidative stress.

The interaction between exosomal miRNAs and APP plays an important role in the pathogenesis of AD. Mir-34a, a miRNA strongly related to cognitive dysfunction, is highly expressed in postmortem brain tissue of AD patients. It has been found that miR-34a promotes amyloid processing of APP, whereas knockdown of miR-34a reduces APP accumulation in brain tissues.^486,487^ Exosomes secreted by miR-34a-overexpressing neurons can be absorbed by adjacent neurons, resulting in the inhibition of target genes in neural network.^488^ Overexpression of miR-34a in AD tissues is correlated with simultaneous inhibition of target genes of synaptic plasticity, oxidative phosphorylation, and glycolysis. Moreover, miR-193b can also target the 3′ untranslated regions of mRNA (UTR) of APP to exert a regulatory role and further influence the progression of AD. The peripheral blood-derived exosomal miR-193b was measured in the normal population, patients with mild cognitive impairment (MCI) and patients with AD, and it was found that miR-193b is significantly lower in patients with MCI and AD than in the control group and its expression level was negatively correlated with Aβ42.^489^

Several research reports have clarified the roles of oxidative stress in the pathogenesis of neurodegenerative diseases,^490–492^ because oxidative stress has been shown to play an important role in enhancing beta-amyloid and tau hyperphosphorylation.^493^ Low concentrations of miR-141-3p have been observed in plasma exosomes from patients with AD.^494^ A large amount of miR-141-3p was also found in the exosomes of astrocytes stimulated by inflammation.^495^ Further studies have confirmed that miR-141-3p can damage the antioxidant defense system and upregulate oxidative stress by inhibiting PTEN.^496^ In addition, exosomal miR-125b-5p is also involved in the process of AD.^497^ Transfection of miR-125b significantly promoted neuronal apoptosis and Tau phosphorylation by activating cyclin-dependent kinase 5 (CDK5) and p35/25.^498^ Besides, inhibition of miR-125b-5p reduces ROS levels, showing a neuroprotective effect against oxidative stress.^499^

Exosomal lncRNAs also take part in the pathogenesis of AD. LncRNA BACE1-AS is upregulated in the brain of patients with AD. The levels of lncRNA BACE1-AS in plasma derived exosomes of AD and healthy individuals have been determined. No significant difference was observed between the two groups. However, in the whole plasma sample, there was significant difference between AD and the control groups.^500^

#### Exosomal ncRNA in PD

PD is characterized by bradykinesia, resting tremor and postural and gait disorders. Typical pathological features of PD are loss of dopaminergic (DAergic) neurons in the dense part of substantia nigra and aggregation of α-SYN protein in Lewy bodies and neurites.^501,502^ Exosomes-derived ncRNAs are involved in the pathophysiology of PD.^503–506^

Multiple miRNAs are reported to be upregulated in exosomes of PD cell model, including miR-210-5p, miR-128-1-5p, miR-505-5p, miR-325-5p, miR-16-5p, miR-1306-5p, miR-669b-5p, miR-125b5p, miR-450b-3p, miR-24-2-5p, and miR-2 -6516-3p and miR-1291. These exosomal miRNAs regulate important pathways in the pathogenesis of PD, such as autophagy, inflammation, and protein aggregation.^507^

Similarly, changes in exosomal miRNAs are found in the cerebrospinal fluid (CSF) of PD patients. Among them, exosomal miR-153, miR-409-3p, miR-10a-5p, and let-7g-3p were significantly increased in CSF of PD patients, while miR-1 and mir19b-3p were significantly decreased.^508^ Let-7 miRNA family is highly conserved in animal species. It has been reported that let-7 was highly expressed in the PD model.^509^ The expression of exosomal let-7 in CSF of PD patients was upregulated.^510^ When exosomal let-7 is absorbed by neurons, it leads to neurodegenerative changes by activating TLR7.^511^ In addition, in *C. elegans* PD model, silence of let-7 decreases the accumulation of α-SYN protein, thereby alleviating the progression of PD.^512^

In addition, the levels of exosomal miRNAs altered in the circulating plasma of PD patients. MiR-195, miR-24, and miR-331-5p were upregulated whereas miR-19b and miR-505 were downregulated in circulating exosomes of patients with PD.^513,514^ Moreover, the increase of miR-137 was observed in the plasma of PD patients.^515,516^ Downregulation of exosomal miR-137 can upregulate oxidative resistance 1 (OXR1) in PD mice model, thereby generating a neuroprotective effect.^517^

When the level of lncRNA extracted from exosomes in plasma samples from PD patients and control group was measured, it was found that 15 and 24 exosomal lncRNAs were upregulated and downregulated, respectively. Among them, lnc-MKRN2-42:1 was positively correlated with MDS-UPDRS III score, which is used to evaluate the severity of dyskinesia in PD patients.^518^ Compared with the control group, the concentrations of lnc-POU3F3 and α-SYN increased in neuro-derived L1CAM exosomes in PD patients. There was a significant correlation between L1CAM exosomal lnc-POU3F3 levels and PD severity, including motor/cognitive impairment.^519^ Four lncRNAs (SNCA-AS1, MAPT AS1, AK127687, and AX747125) were detected in exosomes from human cerebrospinal fluid, providing preliminary evidence that these lncRNAs may be of potential use as a diagnostic tool for PD.^520^ However, further research is needed to elucidate their possible role in PD.

In this chapter, we review recent research on the roles of ncRNAs, especially miRNAs and lncRNAs, in neurodegenerative diseases with an emphasis on AD and PD, whereas exosomal circRNAs have been poorly studied. This fascinating area needs to be explored further.

### The roles of exosomal ncRNA in autoimmune diseases

Autoimmune diseases refer to the diseases caused by the immune response of human body to its own antigens hence causing damage to its own tissues. Rheumatoid arthritis (RA) and systemic lupus erythematosus (SLE) are the most common systemic autoimmune diseases. The pathogenesis of autoimmune diseases is complex and a lot about it is still unknown. The current research studies shows that ncRNAs encapsulated in exosomes play a critical role in autoimmune diseases.

#### Exosomal ncRNA in RA

RA is a chronic autoimmune disease characterized by infiltration of leukocyte into joints, causing production of inflammatory mediators and destruction of bone and cartilage tissue.^521^ Synovitis mediated articular cartilage destruction is associated with upregulation of matrix metalloproteinases (MMPs). This cartilage injury is irreversible and forms a key step in RA joint injury.^522^ Angiogenesis mediates the delivery of nutrients and inflammatory factors. Continuous angiogenesis leads to chronic changes of synovium in RA.^523^ Part of the role of MMPs in regulating vascular remodeling is that they activate the secretion of VGF-β in the stromal matrix by activating VEGF.^524^

MiR-150-5p is associated with T cell maturation and is therefore involved in autoimmune diseases.^525^ It was also confirmed that miR-150-5p could regulate angiogenesis.^526^ Compared with patients with osteoarthritis, there is a decrease in expression of miR-150-5p and an increase in expression of VEGF and MMP-14 as well as angiogenesis in RA patients.^527^ The previous study showed that exosomal micRNA-150 alleviates RA symptoms by downregulating MMP14 and VEGF as well as inhibiting angiogenesis.^527^ In addition, the miRNA microarray analysis showed that miR-548a-3p was significantly reduced in serum exosomes of RA patients. Serum exosomal miR-548a-3p was negatively correlated with serum levels of CRP, rheumatoid factor (RF), and erythrocyte sedimentation rate (ESR) in RA patients. Further research has proven that exosomal miR-548a-3p is involved in the regulation of macrophage mediated inflammation through the TLR4/NF-κB signaling pathway in RA. Therefore, exosomes may be an important factor in predicting RA disease activity.^528^

LncRNAs profiles in RA serum exosomes were analyzed, and it was found that Hotair, LUST, anti-NOS2a, MEG9, SNHG4, TUG1, and NET1 were upregulated in RA serum exosomes and Hotair expression level was increased by an average of about 4 times in RA exosomes. Further studies confirmed that the overexpression of Hotair in RA exosomes may be involved in the pathogenesis of RA in two ways. First, it attracts activated macrophages and induces an immune response. Secondly, Hotair may be involved in the production of MMP in osteoclasts and RA synovial cells.^529^

#### Exosomal ncRNA in SLE

SLE is an autoimmune disease involving multiple systems and organs, which is characterized by persistent inflammation and autoantibody production. MiRNAs carried by exosomes are involved in the pathogenesis of SLE, especially in regulating inflammation and immune imbalance.

Exosomal miRNAs play an important role in the immune-pathophysiology of SLE.^530,531^ Dysregulation of miRNA has been found in SLE, and exosomal miRNA from patients with SLE can regulate inflammation and adaptive immune response.^532^ Previous studies have found that miR-155 promotes autoimmune process by inhibiting suppressor of cytokine signaling-1 (SOCS-1),^533^ or by repressing transcription of PU.1 and TNF-α.^534^ Exosomal miR-155 expression was significantly upregulated in patients with SLE, which was higher in patients with lupus nephritis (LN).^535^ Defects in miR-155 ameliorate autoimmune inflammation in SLE.^536^ Moreover, plasma miR-21 levels in patients with SLE were higher than with healthy controls.^537^ Further studies have confirmed that miR-21 modulates abnormal T-cell responses in SLE patients.^538^ MiR-21 was significantly upregulated in exosomes of SLE patients.^535^ Moreover, exosomal miR-21 from SLE patients can induce the production of type I interferon by dendritic cells.^532^

The expression of serum exosomal miR-451a is correlated with SLE disease activity and renal injury. The level of serum exosome miR-451a in patients with active SLE was significantly lower than that in patients with inactive SLE and control group, especially in those with renal damage. Glucocorticoid or hydroxychloroquine increases the expression of exosomal miR-451a in CD4^+^ T cells.^539^ The aging of MSCs plays an important role in the occurrence and progression of SLE. The expression of miR-146a in serum exosomes of patients with SLE decreases significantly compared with healthy controls. MiR-146a is internalized into MSCs through exosomes and is involved in MSCs senescence by targeting TRAF6/NF-κB signaling pathway.^540^ In addition, it also found that miR-146a was associated with SLE activity and proteinuria.^541^

LN occurs in 40 to 75% of patients with SLE, which is one of the leading causes of death in these patients.^542^ Compared with other biological samples, urine samples are easy to obtain hence less costly. Urinary exosomal miRNAs can accurately reflect renal dysfunction and structural damage.^543,544^ Multiple urinary exosomal miRNAs have been found to be associated with renal fibrosis,^545,546^ and three of these were associated with renal fibrosis in LN, including miR-410, miR-29c, and miR-150.^547–549^ Among them, miR-410 directly interacts with IL-6 3’- UTR to reduce its expression and inhibits renal fibrosis^548^ whereas miR-150 promotes renal fibrosis by downregulating SOCS1.^547^ MiR-29c levels were inversely correlated with histological chronicity index and glomerulosclerosis, but not with renal function.^549^ In addition, let-7 and miR-21 were downregulated in patients with active LN compared with patients with inactive LN. It was reported that let-7 and miR-21 are downregulated during disease flare and upregulated after treatment. It is suggested that urinary exosomal miRNAs (let-7a and miR-21) can be used to guide the clinical staging of patients with LN.^550^

In this section, we discuss the roles of exosomal ncRNAs in autoimmune diseases including RA and SLE. Exosomal miR-150-5p alleviates RA symptoms by regulating VEGF, MMP-14, and angiogenesis, while exosomal miR-548a-3p regulates macrophage-mediated inflammation in RA. Exosomal lncRNAs Hotair, LUST, anti-NOS2a, MEG9, SNHG4, TUG1, and NET1 may be involved in the pathogenesis of RA. In addition, exosomal miRNAs have been implicated in the pathogenesis of SLE, especially in the regulation of inflammation and immune imbalance, activity, and complication, mainly associated with LN.

### The roles of exosomal ncRNAs in infectious diseases

Infectious diseases refer to a group of diseases that are caused by viruses, bacteria, fungi, or other pathogens. Among many infectious diseases, pneumonia and viral hepatitis are very common in the human population. Recently, accumulating evidence revealed that exosomal ncRNAs participated in the pathogenesis of infectious diseases.^551–553^ In this study, we summarize the current evidence about the roles of exosomal ncRNAs in pneumonia and viral hepatitis.

#### Exosomal ncRNAs in pneumonia

Pneumonia is a very common disease worldwide. Severe community-acquired pneumonia remains a life-threatening disease especially in children and elderly.^554^ It has been demonstrated that exosomal ncRNAs levels are markedly different in patients with pneumonia.^553,555^

Exosomal miRNAs are the most studied ncRNAs involved in the development of pneumonia. Recently, it was reported that abundant exosomes selectively loaded with miR-155 are presented in circulation from sepsis-related acute lung injury (ALI) mice.^555^ Injection of exosomes harvested from ALI mice significantly increased the number of M1 macrophages in the lung and led to the inflammation in healthy mice through activating the NF-κB signaling pathway and the downstream upregulation of TNF-α and IL-6. Besides, circulating exosomal miR-155 was also found to promote macrophage proliferation and inflammation by regulating SHIP1 and SOCS1.^555^ According to Quan et al. the expression of exosomal miR-371b-5p derived from injured alveolar progenitor type II cells is upregulated and decreases the expression of PTEN.^556^ This promotes cell proliferation and re-epithelialization of injured alveoli through activating phosphorylation of Akt and its substrates GSK3β and FOXOs. Importantly, the elevated levels of exosomal miR-371b-5p were also observed in lavage samples from patients with acute pneumonia, which may provide a potential therapeutic target for acute pneumonia.

Dysregulated immune reaction is a major contributing factor to the pathophysiology of pneumonia and is associated with the high incidence ratio of pneumonia. It was found that exosomal miR-221 and miR-222 levels derived from lipopolysaccharide (LPS)-induced macrophage increases and promotes the proliferation of epithelial cells.^557^ Besides, macrophages induced by LPS also releases high number of exosomes that deliver miR-223 and miR-142 hence inhibiting the LPS-triggered inflammatory responses in the lung.^558^ However, the expression of exosomal miR-103a-3p from LPS-induced lung cells and from circulation of pneumonia patients were decreased. Overexpression of exosomal miR-103a-3p attenuates inflammation by regulating immune response via transducing β-like 1X related protein 1/NF-κB signaling pathway.^559^

#### Exosomal ncRNAs in viral hepatitis

Viral hepatitis is an infectious disease caused by a variety of hepatitis viruses. If not treated, it can develop into liver cirrhosis or even liver cancer which is very dangerous for human health. Therefore, exploration of the underlying mechanisms of viral hepatitis is of great significance. Accumulating evidence demonstrates that exosomal ncRNAs are involved in the pathogenesis of viral hepatitis.^560^

Exosomal miRNAs has been demonstrated to be involved in the regulation of multiple signaling pathways during the development of viral hepatitis.^561,562^ Globally, hepatitis B virus (HBV) is the most common hepatitis and causes very heavy economic and heathy burden. According to Li et al. some exosomal miRNAs, such as miR-221-3p and miR-25-3p are upregulated in patients with chronic HBV infection, whereas other such as miR-372-3 and miR-10a-5p are downregulated.^563^ Besides, miR-122, miR-204, miR-let7c, miR-23b, and miR150 are also altered and are correlated with HBV-related liver diseases.^562^

Hepatitis C is also common in the human population. The fibrosis of the liver is correlated with the chronic infection of hepatitis C virus (HCV). However, the specific mechanisms remain poorly understood. Recently, Devhare et al. reported that the exosomal miR-19a released by HCV-infected hepatocytes is upregulated and increases the expression of fibrosis marker genes in hepatic stellate cells (HSC).^564^ Further analysis revealed that the exosomal miR-19a aggravates the liver fibrosis by activating the SOC3/STAT3/TGF-β signaling pathway.

Relatively, there are few studies that evaluated exosomal lncRNAs in the development of viral hepatitis.^565^ It was found that the expression of runt-related transcription factor 1 (RUNX1) and RUNX1 overlapping RNA (RUNXOR) are significantly upregulated in myeloid-derived suppressor cells (MDSCs) during chronic HCV infection. These were positively correlated with the immunosuppressive molecule levels.^565^ Mechanistically, it has been demonstrated that HCV-associated exosomes deliver RUNXOR and RUNX1 to MDSCs and promote the function of differentiation and suppressive functions of MDSCs through regulation of STAT3 signaling pathway. These results indicate that RUNXOR and RUNX1 may become a promising target for immunomodulation with antiviral treatment during HCV infection.

In summary, the roles of exosomal miRNAs in pneumonia has been studied most intensively, among which miR-155 and miR-371B-5p are related to the occurrence and development of pneumonia, while miR-221, miR-222, miR-223, miR-142, and miR-103a-3p are associated with the immune response of pneumonia. As for viral hepatitis, exosomal miRNAs have been shown to regulate multiple signaling pathways, whereas few exosomal lncRNAs (RUNXOR and RUNX1) have been demonstrated to be involved in the development of viral hepatitis.

## Clinical applications of exosomal ncRNAs in human diseases

Recently, exosomal ncRNAs have emerged as novel players in the occurrence and development of various human diseases. Circulating ncRNA profiles in exosomes are altered for a wide range of diseases, suggesting that exosomal ncRNAs may serve as cycling indicators of an individual’s physiological status and targeted therapies tools in precision medicine. Therefore, exosomal ncRNAs exert potential to be candidate biomarkers and therapeutic targets for the diseases. This section summarizes the present knowledge on the potential clinical implication of exosomal ncRNAs in biomarker identification and therapy exploration in diseases.

### Exosomal ncRNAs as diagnostic biomarkers in human diseases

Biomarker are a series of molecules that can be utilized for disease detection and/or prognosis prediction. Sensitivity, specificity, stability, and relatively non-invasive are most important and necessary characteristics of a good biomarker. Notably, emerging research is targeting exosomes as potential biomarkers because they can be detected using simple and inexpensive methods in many body fluids and are stable against enzyme degradation by providing a protective transportation system. It is worth noting that exosomal ncRNAs are differentially expressed in human lifestyle activities and various pathological diseases, including cardiovascular, metabolic, neurodegenerative, autoimmune, infectious, and cancerous disorders, which underlies the potential of ncRNAs as promising biomarkers for early detection.^360,364,566–569^ This study compiles the circulating exosomal ncRNAs that have been reported to be altered during the course of human diseases, as detailed in Table 1.

**Table 1:** Exosomal ncRNAs as potential diagnostic biomarkers in human diseases

Diseases	Cargoes	Expression	Effects	Refs
Lung cancer	miR-23a	↑	Inducing phenotypic changes, increasing vascular permeability and cancer migration	^137,150^
	miR-126	↑	Inducing loss of malignancy of NSCLC cells	^144^
	miR-193a-3p, miR-210-3p, miR-5100	↑	Activating STAT3 signaling and increasing the expression of mesenchymal related molecules	^138^
	miR-499a-5p	↑	Promoting cell proliferation, migration and EMT	^139^
	GAS5	↑	Function as an ideal noninvasive biomarker for NSCLC	^154^
	SOX2-OT	↑	Serving as a promising non-invasive plasma-based tumor biomarker for LSCC	^155^
	circSATB2	↑	Acting as a biomarker for the diagnosis of NSCLC	^156^
	circ-0007761, circ-0047921, circ-0056285	↑	Acting as promising biomarkers for NSCLC diagnosis	^159^
BC	miR-9	↑	Inducing tumor growth	^174^
	miR-10b	↑	Modulating tumor microenvironment	^170^
	miR-1246	↑	Regulating breast tumor progression	^171^
	miR-20a-5p	↑	Inducing proliferation and differentiation of osteoclasts	^183^
	miR-134	↓	Acting as a potential biomarker for BC	^172^
	MALAT1	↑	Inducing BC progression	^188^
	H19	↑	Acting as a biomarker for the diagnosis of BC	^190^
	circHIF1A	↑	Regulating stem cell properties of BC	^196^
HCC	miR-10b-5p	↑	Acting as a biomarker for early-stage HCC	^211^
	miR-125b	↓	Associated with tumor number, encapsulation, and TNM stage	^214^
	miR-18a, miR-221, miR-222, miR-224	↑	Function as novel serological biomarkers for HCC	^217^
	miR-101, miR-106b, miR-122, miR-195	↓		
	FAL1	↑	Promoting cell proliferation and metastasis	^220^
	H19	↑	Promoting cell proliferation, migration, and invasion	^221^
	LINC00161	↑	Serving as a novel biomarker for HCC	^227^
	HEIH	↑	Serving as a novel biomarker for HCC	^230^
	SENP3-EIF4A1	↓	Inducing tumor growth	^231^
	FAM138B	↓	Inhibiting HCC growth	^232^
	circUHRF1	↑	Inducing immunosuppression	^240^
CRC	miR-1229, miR-1246, miR-150, miR-21, miR-223, miR-23a	↑	Promising biomarkers for non-invasive diagnosis of CRC	^256^
	miR-23a, miR-301a	↑	Promising biomarkers for non-invasive diagnosis of CRC	^257^
	miR-6803-5p	↑	Serving as a diagnostic and prognostic biomarker	^259^
	LINC02418	↑	Inducing tumorigenesis	^267^
	NNT-AS1	↑	Inducing the proliferation, migration and invasion of CRC cells	^578^
	LINC02418	↑	Involvement in CRC tumorigenesis	^267^
	CCAT2	↑	Serving as a novel potential predictor in CRC	^274^
	LNCV6-116109, LNCV6-98390, LNCV6-38772, LNCV-108266, LNCV6-84003, LNCV6-98602	↑	Serving as potential non-invasive biomarkers for early diagnosis of CRC	^275^
	circPACRGL	↑	Inducing cell proliferation, migration and invasion	^278^
	circFMN2	↑	Mediating cell proliferation and migration	^279^
	hsa-circ-0004771	↑	Serving as a novel diagnostic biomarker of CRC	^283^
GC	miR-34	↓	Inducing GC cell proliferation and invasion and tumor growth	^289^
	miR-1246	↑	Serving as potential biomarker for the early diagnosis of GC	^296^
	miR-1290	↑	Inducing GC cell proliferation and invasion	^285^
	UEGC1	↑	Acting as a promising biomarker in the development of GC	^305^
	GC1	↑	Serving as a noninvasive biomarker for detecting early-stage GC	^307^
	lnc-SLC2A12-10:1	↑	Serving as a noninvasive biomarker for the diagnosis of GC	^310^
	CEBPA-AS1	↑	Promoting cell proliferation, inhibiting apoptosis, and inducing GC progression	^311^
	lnc-GNAQ-6:1	↓	Serving as a potential biomarker for the detection of GC	^312^
	circNEK9	↑	Promoting the proliferation, migration, and invasion of GC cells	^316^
	circ29	↑	Involved in the occurrence and development of GC	^317^
	has-circ-0065149	↓	Acting as a novel biomarker for diagnosis	^320^
PCa	miR-141	↑	Function as a useful biomarker for the diagnosis of metastatic PCa	^331^
	circ-0044516	↑	Inducing tumor cell metastasis	^337^
T2DM	miR-20b-5p	↑	Attenuating insulin-stimulated glycogen accumulation	^363^
Obesity	miR-26a	↓	Inversely correlated with BMI	^360^
MetS	MALAT1	↓	Serving as a potential epigenetic biomarker of diabetes risk or MetS	^366^
OP	miR-214	↑	Repressing osteoblasts activity	^395^
	hsa-circ-0006859	↑	Serving as a high sensitivity and specificity biomarker	^404^
Hypertension	miR-155-5p	↓	Promoting VSMCs proliferation and vascular remodeling	^422^
	miR-27a	↑	Promoting vasodilation and causing hypertension	^423^
	miR-425-5p, miR-17-5p	↑	Potentially serving as biomarkers	^421^
	miR-17	↓	Promoting ECs inflammation	^424^
AS	miR-223	↑	Inhibiting inflammation and VSMCs proliferation	^434,435^
	miR-92a	↑	Promoting ECs inflammation and the formation of atherosclerotic plaque	^437^
	miR-342-5p	↑	Promoting H_2_O_2_-induced ECs apoptosis	^438^
	miR-155	↑	Inhibiting ECs proliferation and migration	^430^
	miR-21-3p	↑	﻿Promoting VSMCs proliferation and migration	^432^
	miR-106a-3p	↑	Promoting proliferation and inhibiting apoptosis in VSMCs	^439^
	GAS5	↑	Promoting the apoptosis of macrophages and ECs	^440^
	MALAT1	↑	Promoting the M2 macrophage polarization, the formation of neutrophil extracellular traps in neutrophils	^442,443^
	circRNA-0006896	↑	Promoting proliferation and migration of ECs	^445^
AMI	miR-125b	↑	Ameliorating cardiomyocytes apoptosis and facilitating ischemic cardiac repair	^450^
	miR-22	↑	Inhibiting apoptosis of cardiomyocytes	^452^
	miR-301	↑	Inhibiting myocardial autophagy	^454^
	miR-25-3p	↑	Reducing myocardial apoptosis and inflammation	^458^
	miR-144	↑	﻿Ameliorating hypoxia-induced cardiomyocyte apoptosis	^459^
	miR-146a	↑	Inhibiting myocardial apoptosis, inflammatory response, and fibrosis	^461^
	MALAT1	↑	Enhancing neovascularization	^448^
	H19	↓	Reducing infarct size and cardiomyocyte apoptosis	^449^
	lncAK139128	↓	Promoting the apoptosis and inhibiting the proliferation, migration, and invasion of cardiac fibroblasts	^463^
HF	miR-425, miR-744	↓	Protecting against myocardial remodeling	^466^
AF	miR-320d	↓	Promoting viability and inhibiting apoptosis of cardiomyocytes	^469^
	miR-107	↑	Suppressing viability and migration of ECs, enhancing cell apoptosis	^470^
AD	miR-34a	↑	Promoting amyloid processing of APP	^486,487^
	miR-141-3p	↑	Damaging the antioxidant defense system and up-regulating oxidative stress	^496^
	miR125b-5p	↑	Promoting neuronal apoptosis and Tau phosphorylation	^498^
PD	let-7	↑	Leading to neurodegenerative changes	^511^
RA	miR-548a-3p	↓	Regulating macrophage mediated inflammation	^528^
	Hotair	↑	Inducing an immune response	^529^
SLE	miR-155	↑	Regulating autoimmune inflammation in SLE	^536^
	miR-21	↑	Inducing the production of type I interferon	^532^
Pneumonia	miR-155	↑	Promoting inflammation and proliferation	^555^
	miR-371b-5p	↑	Promoting proliferation and re-epithelialization of injured alveoli	^556^
	miR-221/222	↑	Promoting epithelial cell proliferation	^557^
	miR-223/142	↑	Inhibiting LPS-induced lung inflammation	^558^
	miR-103a-3p	↓	Inhibiting inflammation	^559^
Viral hepatitis	miR-221-3p, miR-25-3p	↑	Associated with liver fibrosis and inflammation	^563^
	miR-372-3, miR-10a-5p	↓	Associated with liver fibrosis and inflammation	^563^
	miR-19a	↑	Promoting liver fibrosis	^564^

*NSCLC* non-small cell lung cancer, *STAT3* signal transducer and activator of transcription 3, *EMT* epithelial-mesenchymal transition, *LSCC* lung squamous cell carcinoma, *BC* breast cancer, *HCC* hepatocellular carcinoma, *CRC* colorectal cancer, *GC* gastric cancer, *PCa* prostate cancer, *T2DM* type 2 diabetes mellitus, *BMI* body mass index, *MetS* metabolic syndrome, *OP* osteoporosis, *AS* atherosclerosis, *VSMCs* vascular smooth muscle cells, *ECs* endothelial cells, *AMI* acute myocardial infarction, *HF* heart failure, *AF* atrial fibrillation, *AD* Alzheimer’s disease, *APP* amyloid precursor proteins, *PD* Parkinson’s disease, *RA* rheumatoid arthritis, *SLE* systemic lupus erythematosus, *LPS* lipopolysaccharide

### Exosomal ncRNAs-based therapeutics in human diseases

**Fig. 7:**
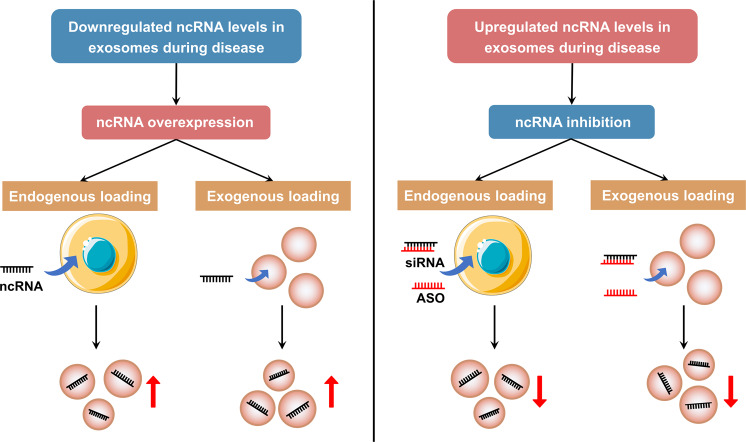
Exosomal ncRNA-based therapeutics in human diseases. Endogenous (pre-loading cargoes into donor cells followed by exosomal cargo release) or exogenous (directly loading cargoes into exosomes after their production or isolation) loading of ncRNAs or their inhibitors into exosomes exhibits significant therapeutic potential. ASO antisense oligonucleotide. This figure was created with the aid of Servier Medical Art (https://smart.servier.com/)

Considering the various advantages of exosomes, such as natural availability, considerate biocompatibility, biological barrier permeability, low immunogenicity, and toxicity, exosomes are poised to become a promising tool for therapeutic vehicles.^7,570,571^ Genetic engineering has been explored to modify exosomes for therapeutic utilization. The ability to modify isolated and purificatory exosomes loaded with mimics or inhibitors of ncRNAs and target molecules on their surface, offers the possibility of using exosomes as vectors to transport specific ncRNAs to specific tissues or organs (Fig. 7). This gene regulation proposes exosomal ncRNAs as promising approach for the treatment of various human diseases.

#### Therapies that upregulate exosomal ncRNAs

Exosomes can load ncRNAs release of which may present promising therapeutic effects. Several studies have explored important therapeutic interventions of exosome-based ncRNAs. By modifying exosomes with ncRNA mimics or inhibitors, exosomes can serve as delivery system of specific ncRNA sequences.

Elevated expression levels of beneficial ncRNAs carried by exosomes have been described to have various therapeutic implications. The levels of ncRNAs in exosomes will enhance by loading ncRNAs into exosomes endogenously or exogenously. Exogenous loading is performed after exosome production or isolation with cargo of interest encapsulated to exosomes by co-incubation, chemical transfection, or physical approaches such as electroporation and dialysis.^572^ For example, Lv et al. used electroporation to package miR-21-5p mimics into ADSC-derived exosomes. The results of their study suggested that the miR-21-5p mimics-enriched exosomes accelerate diabetic wound healing in vivo through promoting re-epithelialization, collagen remodeling, angiogenesis, and vessel maturation.^573^

In addition, cargoes can be loaded in an endogenous way by pre-loading donor cells with therapeutic ncRNA, which will then be encapsulated in exosome. For instance, Zhuo et al. transfected lncRNA FAM138 into HCC cells and then created exosomes enriched in FAM138, which alleviated the progression of HCC by regulating miR-765.^232^ Therefore, endogenous or exogenous loading of beneficial ncRNAs into exosomes exhibits significant therapeutic potential.

#### Therapies that downregulate exosomal ncRNAs

It may also be therapeutic to decrease the amount of detrimental ncRNAs in exosomes. This effect can be achieved through endogenous or exogenous loading of small interfering RNAs (siRNAs) or ncRNA inhibitors such as antisense oligonucleotides (ASO) into exosomes. For instance, exosome-delivered circRNA has-circ-0005963 promotes drug resistance in CRC, whereas siRNA of has-circ-0005963 transported by exosomes can reverse resistance in vivo.^281^ Moreover, miR-21 is an oncogenic miRNA, while delivery of anti-miRNA oligonucleotides miR-21 using modified exosomes can reduce miR-21 level in the glioblastoma, contributing to reduction of tumor size.^574^

Above all, these studies provide new strategies for potential clinical diagnosis and therapeutic management for exosome-mediated human diseases. Exosome-based delivery of curative ncRNAs, as well as siRNAs or inhibitors of harmful ncRNAs has the potential to be used in development of disease therapies. However, the transition of exosomal ncRNAs from basic laboratory research to clinical application remains challenging. Further development of biomedical materials and molecular targeted therapies is needed to elucidate the functions of exosomal ncRNAs.

## Perspectives and conclusions

Knowledge of the roles of exosomal ncRNAs in physiological and pathological conditions has advanced considerably over the past few decades. In this review, we discuss the structures of exosomes and their cargo ncRNAs, and compile the underlying regulatory mechanisms. In order to distinguish between ncRNAs in exosomes and non-exosomes in health and disease, we also strive to study the differences in physiological homeostasis and pathological processes. Exosomes exert their roles in the pathophysiology of multiple disorders by modulating immune response, oxidative stress, autophagy, gut microbe, and cell cycle dysregulation. The impact of exosomes on cell biology is greater than originally expected, which makes related research rather complex. The temporal and spatial expression patterns, precise roles and mechanisms of specific miRNAs, lncRNAs, and circRNAs encapsulated in exosomes remain largely unknown in different system development and diseases.

Interest in the contribution of exosomal ncRNAs to the progression of various diseases, including cancers, metabolic diseases, neurodegenerative diseases, cardiovascular diseases, autoimmune diseases, and infectious diseases, is booming. Tissue-specific alterations of exosomal ncRNAs play an important role in the initiation and development of human diseases as well as their complications. First, exosomal ncRNAs are involved in pathological cellular processes including cell proliferation, migration, invasion, metastasis, angiogenesis, and EMT associated with diverse cancers. Second, exosomal ncRNAs promote cell-to-cell and tissue-to-tissue crosstalk in an autocrine, paracrine or endocrine manner, and hence applying pleiotropic activities in metabolic diseases such as T2DM, obesity, and primary osteoporosis. Third, exosomal ncRNAs serve as regulators of dyslipidemia and myocardial structure and function in cardiovascular diseases including hypertension, AS, AMI, HF, and AF. Fourth, Exosomes carry ncRNAs to regulate communication between neurons and act as mediators involved in the pathogenesis and progression of neurodegenerative diseases. Fifth, exosomal ncRNAs modulate autoimmune diseases mainly by regulating inflammation and immune imbalance. Besides, the roles of exosomal ncRNAs in infectious diseases have been shown to be involved in the development of pneumonia and viral hepatitis. However, much effort remains to be done to understand the full extent of exosomal ncRNAs exerting their pathological effects.

Although exosomal ncRNAs have been identified for a relatively short time, there have been massive progression in clinical and therapeutic applications. In the field of diagnostics, it is noteworthy that exosomal ncRNAs are differentially expressed in human lifestyle activities and a variety of pathological diseases, suggesting that exosomal ncRNAs have the potential to be biomarkers for early detection. Exosomal ncRNAs exert advantages as biomarkers over non-exosomal ncRNAs. NcRNAs harbored in exosomes can be protected from degradation by RNase thus increasing their stability. Exosomal ncRNAs can present a high concentration in body fluid.^575^ Several studies have compared exosomal and non-exosomal miRNAs as biomarkers, and 75% of the studies reported that exosomal pattern of miRNAs are more important in biological processes compared with non-exosomal ones. This can be attributed to the advantages of exosomal miRNAs including quantity, quality, and stability.^576^ In the area of therapeutics, the use of exosomal ncRNAs have many potential benefits, so increasing exosomal ncRNAs therapies will enter a formal drug development process. Exosomes bearing a specific cargo can function as a drug delivery system. Exosomes have several advantages when delivering ncRNAs compared with traditional delivery methods, such as virus and liposome. Use of exosomes as endogenous vehicles can evade immune response, thus minimizing their toxicity. An in vivo study conducted by Mendt et al. reported no side effects including adverse immune response even after repeated administration of exosomes.^570^ Furthermore, exosomes can target specific tissues and cells through specific ligands on the surface.^577^ Moreover, exosomes can readily penetrate biological barriers such as the blood-brain barrier (BBB). For instance, an in vivo study reported that exosomes can cross the BBB and deliver siRNAs into the brain.^571^ Despite exosomal ncRNAs have great potential as biomarkers and therapeutics for a wide range of human diseases, there are many hurdles to bring them to clinic. First of all, we need to determine the extent to which purified exosomes are likely to be sufficient to confer the positive effects. Secondly, the dynamics and pharmacokinetics, as well as toxic studies of potential exosomal ncRNAs drugs require to be repeatedly tested. Besides, there is urgent need for experiments using animal and clinical models to identify whether exosomal ncRNAs exert regulatory homeostasis or pathological functions. Overcoming these obstacles will take the field to another unprecedented level.

The purpose of this study is to explore and strengthen the understanding of the mechanisms and roles of exosomal ncRNAs in human health and diseases, and to provide the basis for new clinical diagnosis and therapy strategies.
